# Recent Progress in Nanomaterial-Based Biosensors and Theranostic Nanomedicine for Bladder Cancer

**DOI:** 10.3390/bios13010106

**Published:** 2023-01-06

**Authors:** Fan-Xin Song, Xiaojian Xu, Hengze Ding, Le Yu, Haochen Huang, Jinting Hao, Chenghao Wu, Rui Liang, Shaohua Zhang

**Affiliations:** 1 Department of Urology, The Third Affiliated Hospital of Shenzhen University, Shenzhen University, Shenzhen 518000, China; 2 Institute of Functional Nano & Soft Materials (FUNSOM), Soochow University, Suzhou 215123, China; 3 College of Nano Science & Technology (CNST), Soochow University, Suzhou 215123, China; 4 Department of Urology, The First Affiliated Hospital of Soochow University, Suzhou 215006, China; 5 Department of Urology, The Affiliated South China Hospital of Shenzhen University, Shenzhen University, Shenzhen 518000, China

**Keywords:** bladder cancer, nanomedicine, diagnosis, therapy, biosensors, drug delivery systems

## Abstract

Bladder cancer (BCa) is one of the most expensive and common malignancies in the urinary system due to its high progression and recurrence rate. Although there are various methods, including cystoscopy, biopsy, and cytology, that have become the standard diagnosis methods for BCa, their intrinsic invasive and inaccurate properties need to be overcome. The novel urine cancer biomarkers are assisted by nanomaterials-based biosensors, such as field-effect transistors (FETs) with high sensitivity and specificity, which may provide solutions to these problems. In addition, nanomaterials can be applied for the advancement of next-generation optical imaging techniques and the contrast agents of conventional techniques; for example, magnetic resonance imaging (MRI) for the diagnosis of BCa. Regarding BCa therapy, nanocarriers, including mucoadhesive nanoparticles and other polymeric nanoparticles, successfully overcome the disadvantages of conventional intravesical instillation and improve the efficacy and safety of intravesical chemotherapy for BCa. Aside from chemotherapy, nanomedicine-based novel therapies, including photodynamic therapy (PDT), photothermal therapy (PTT), chemodynamic therapy (CDT), sonodynamic therapy (SDT), and combination therapy, have afforded us new ways to provide BC therapy and hope, which can be translated into the clinic. In addition, nanomotors and the nanomaterials-based solid tumor disassociation strategy provide new ideas for future research. Here, the advances in BCa diagnosis and therapy mentioned above are reviewed in this paper.

## 1. Introduction

As a complicated disease correlated with high morbidity and mortality without optimal treatment, bladder cancer (BCa) is one of the most common malignant diseases in the urinary system with an estimated 81,180 new cases and 17,100 deaths in the United States in 2022 [1,2]. The presentation of BCa can be categorized into muscle-invasive bladder cancer (MIBC), non-muscle-invasive bladder cancer (NMIBC), or metastatic forms of the disease according to the different molecular drivers [3]. Among bladder cancer patients, nearly 80% of cases are NMIBC at the initial diagnosis with a probability of about 70% for a recurrence and about 15% for progression following standard treatment, which is based on the clinical guidelines. In addition, 25% of cases are MIBC (T2a–T4b) and most of the patients with MIBC have a poor prognosis and primary invasive bladder cancer [4]. The cost for patients is magnified by the multiple tests and treatments, which are due to the high recurrence and progression of bladder cancer [5]. According to morphology, bladder cancer can be grouped into solid, papillary, and mixed types [2].

The majority of bladder cancer patients are diagnosed during the test prompted by hematuria, which is one of the symptoms that is strongly correlated with bladder cancer [6]. Despite the hematuria, bladder cancer is also followed by frequent painful urination and pelvic pain [7]. After the symptoms are correlated with bladder cancer, urinary cytology and cystoscopy can be applied as the first-line approach to reach a diagnosis. The modification of the cystoscopy used for sample collection for biopsy determination can also be realized. Furthermore, to examine the severity, diagnostic techniques including magnetic resonance imaging (MRI), computed tomography (CT) scan, positron emission tomography (PET), ultrasound imaging, bone scan, and chest X-ray can be exploited [8]. However, the diagnosis of bladder cancer is challenging due to the high cost, invasive property, low sensitivity, or low specificity of traditional diagnostic techniques. Thus, non-invasive techniques, such as novel optical imaging techniques and urine-based tests with high sensitivity and specificity are being developed to assist in the diagnosis. The management of bladder cancer varies according to the grade and type of bladder cancer [7,9]. Intravesical instillation of pharmaceuticals for chemotherapy or immunotherapy after transurethral resection of the bladder tumor (TURBT) is a common choice for the adjuvant therapy of NMIBC [10], and it has been confirmed to eradicate the residual cancer cells effectively after surgery to avoid recurrence [11]. The combinatorial drugs in the form of gemcitabine and paclitaxel (GP), gemcitabine and cisplatin (GC), methotrexate, cisplatin, and vinblastine (CMV) can be utilized for bladder cancer chemotherapy via systemic or intravesical administration [8,12] For the immunotherapy of bladder cancer, intravesical administration of the *bacillus Calmette-Guerin (BCG)* vaccine can mediate the immune reactions [13]. In addition, various other immunotherapy drugs can be used via intravenous administration [14]. In the case of MIBC, treatments including neoadjuvant therapy, radiotherapy, partial cystectomy, or radical cystectomy can be utilized [15]. Similarly, there are also some shortcomings of conventional treatments that need to be overcome. Although the clinical interventions can alleviate tumor progression and recurrence, many patients still deteriorate into metastatic disease and suffer a poor prognosis [15]. 

Nanotechnology is the “intentional design, characterization, production, and applications of materials, structures, devices, and systems by controlling their size and shape in the nanoscale range (1 to 100 nm)” [16]. Because of the similarities in scale between biological molecules and nanomaterials and the capabilities of nanosystems that can be manipulated into various functions, nanotechnology has potential in the medical field. Nanomedicine aims to apply the physical characteristics and properties of nanomaterials to solve the problems presented in the diagnosis and treatment of diseases [16]. Compared with atoms and macroscopic materials, nanomaterials have unique tunable electronic, optical, magnetic and biological properties. Nanodrug systems have been applied for the improvement of safety, efficacy, the pharmacokinetic and pharmacodynamic profile, and the physicochemical properties of drugs [17]. Take the example of paclitaxel (PTX), the nanoparticle albumin-bound (nab)-paclitaxel has been demonstrated to be more efficient and tolerated compared with the conventional PTX which not only has poor solubility in water but causes a large systemic adverse reaction after intravenous administration [18,19]. Nab-paclitaxel has also been indicated to have a better efficacy on BCa (Table 1) [20,21]. In addition, nanomaterials can be engineered to aid in highly sensitive and high-throughput diseases biomarker detection, high-quality biomedical imaging, and the precise delivery of therapeutic agents for BCa diagnosis and therapy. 

**Figure 1:**
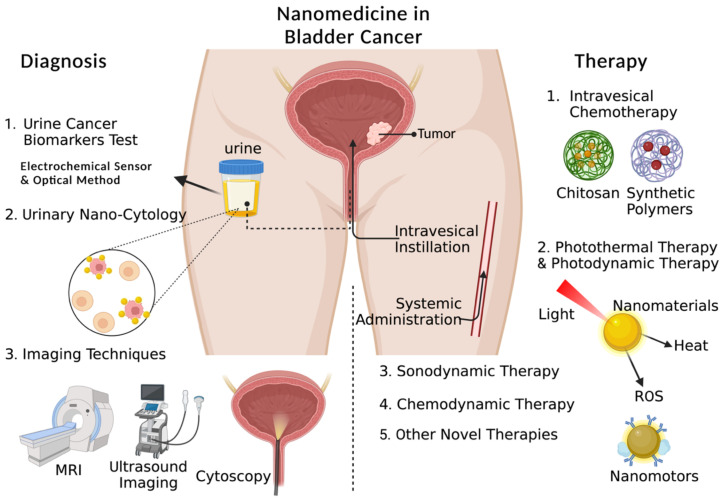
Schematic illustration of nanomedicine in bladder cancer diagnosis and therapy (Created with BioRender.com, access date: 10 December 2022).

In this review, we summarize not only the nanomaterials-based high-performance biosensors, including electrochemical and optical methods for urinary cancer biomarker detection, but also nano-cytology and next-generation imaging techniques for BCa (Figure 1). Concerning bladder cancer therapy, since intravesical instillation has been standard therapy for bladder cancer, various nanocarriers have been intensively applied for drug delivery for the improvement of intravesical instillation and to provide solutions to the shortcomings of conventional intravesical instillation. In addition, the delivery of macromolecular drugs has been realized with the assistance of nanocarriers. Except for intravesical chemotherapy, other novel nanomedicine-based therapies, including photothermal therapy (PTT), photodynamic therapy (PDT), sonodynamic therapy (SDT), chemodynamic therapy (CDT) and combination therapy, have been widely utilized for bladder cancer therapy. Nanomotors and nanomaterials-based solid tumor disaggregation strategies also take part in novel therapies for bladder cancer, which provides new ideas for future research (Figure 1). This review aims to afford a relatively comprehensive summary of the advance in the application of nanomedicine in bladder cancer diagnosis and therapy. 

## 2. Nanomaterial-Based Bladder Cancer Diagnosis

### 2.1. Urine Cancer Biomarker Test

Traditional bladder cancer diagnostic approaches have some disadvantages. Cystoscopy [22] and biopsy [23] are invasive and painful. Although urine cytology is widely applied as a gold standard diagnostic technique for its advantages of being non-invasive, non-expensive, easy to perform, and a high specificity, which is up to 98% in high-grade bladder cancer detection, the sensitivity of urine cytology is relatively low (less than 40%), suggesting that it is not perfect enough to be a primary evaluation diagnostic tool [24].

A biomarker is “a characteristic that is objectively measured and evaluated as an indicator of normal biological processes, pathogenic processes, or pharmacological responses to a therapeutic intervention” [25]. Urine cancer biomarker tests can be used not only in diagnosis but in recurrence monitoring and treatment response measurements [24]. The higher sensitivity and comparable specificity of the urine cancer biomarker test, has more potential than cytology and may even replace it [26]. In addition, urine cancer biomarker detection enables the improvement of the biosafety of bladder cancer diagnostic techniques. It has been shown that several commercial urine cancer biomarker test kits have been approved by the European Conformity Approval (CE) or the U.S. Food and Drug Administration (FDA) [24]. Nowadays, nanotechnology has been applied for the development of next-generation techniques which have a miniaturization platform, higher sensitivity, multiplex detection ability, or real-time monitoring ability [27]. Here, we will discuss the different nanomaterial-based techniques, which have been developed for urine cancer biomarker detection and bladder cancer diagnosis, as we believe that the development of these point-of-care urine biomarker detection techniques for the diagnosis of bladder cancer will finally be exploited in the clinic and benefit a wide range of people. 

#### 2.1.1. Nanomaterial-Based Electrochemical Sensors

The enzyme-linked immunosorbent assay (ELISA) is the most widely applied clinical protein biomarker detection technique [28]; however, its utilization in the point-of-care (POC) diagnosis is limited due to the relatively expensive test kit and the requirement for bulky plate readers. Similarly, the commercial multiplexed protein automated or semiautomated techniques, which may employ fluorescence (Luminex, Myriad RBM), surface plasmon resonance (Horiba Inc., BIO-RAD), or electrochemiluminescence (Roche Diagnostic, Mesoscale Discovery), are limited for POC diagnostic applications because of their expensive instruments and specialized consumables [29]. Electrochemical sensors that utilize nanomaterials not only have improved sensitivity and accuracy within the urine cancer biomarker test [30], but have the advantages of being simple to operate and low cost, which provides them with the potential to be applied in clinics [29]. The parameters of these amperometric sensors, reported for BCa urine biomarker detection, are summarized in Table 2, and the parameters of those Bio-FETs reported for BCa urine cancer biomarker detection are summarized in Table 3.

For the practical application of nanomaterial-based biosensors, both their performance and cost should be considered. Two of the main factors considered when evaluating a biosensor are sensitivity and selectivity. The limit of detection (LOD) is an important parameter for the assessment of sensitivity. For selectivity, not only should the concentration of the biomarkers in the urine sample of BCa patients be significantly different from that of healthy donors, but the biosensors should be able to avoid false-negative and/or false-positive results caused by the complex components contained in the urine sample. Although the antibodies applied can ensure the performance of biosensors, they make the biosensors relatively expensive. In addition, the barriers to mass production of these biosensors should also be overcome in future.

##### Electrochemical ELISA

**Figure 2:**
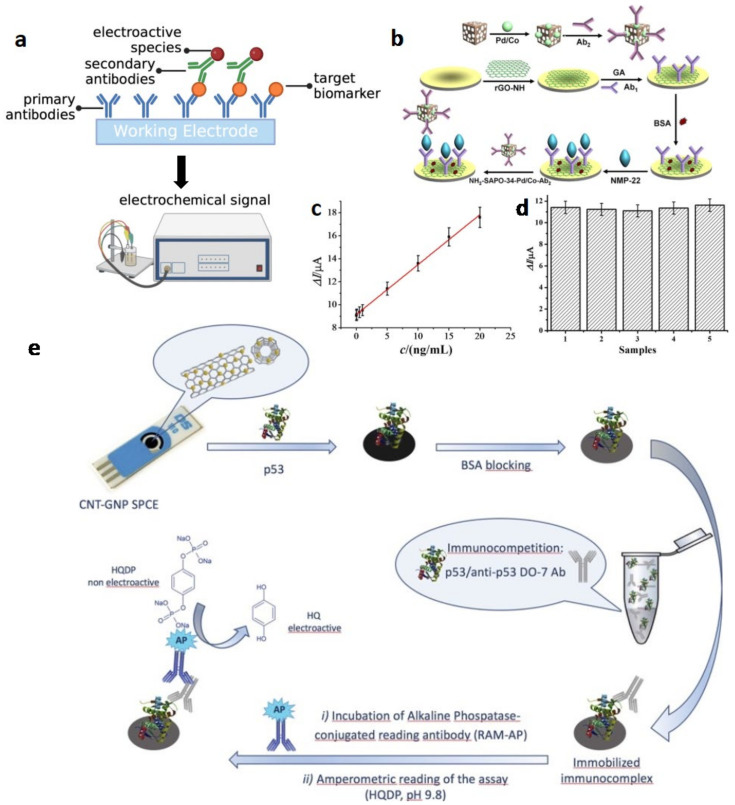
(**a**) Schematic of the sandwich-based model of the eELISA (Created with biorender.com, access date: 10 December 2022). (**b**) Schematic of the construction of the NH_2_-SAPO-34-Pd/Co-Ab_2_ and the eELISA sensor for urine cancer biomarker detection. (**c**) Calibration curve of the eELISA sensor toward different concentrations of NMP-22. (**d**) Amperometric response of the eELISA sensor to pure NMP-22 and NMP-22 with interfering substances (from left to right: NMP-22, NMP-22 + BSA, NMP-22 + vitamin C, NMP-22 + trioxypurine, NMP-22 + glucose). (Reprinted with permission Ref. [33]. Copyright © The Author(s)). (**e**) Schematic of the fabrication of the CNT/GNP SPCE competitive immunosensor. (Reprinted with permission Ref. [35]. Copyright © 2017 Elsevier B.V. All rights reserved).

Electrochemical ELISA (eELISA) is an integration of the ELISA assay and the electrochemical voltametric and amperometric sensor; it turns the concentration of analytes into an electrochemical signal. It has been proposed to overcome the limitation of the laborious procedure and relatively high sample volume of conventional optical ELISA and further improve the LOD so that it is more suitable for early-stage diagnosis [31]. The majority of the present eELISA techniques operate in a sandwich-based model [32]. Electrochemical sandwich ELISA is a kind of electrochemical immunoassay [31], which works as follows (Figure 2a): firstly, a target biomarker is recognized by the primary antibody (Ab_1_) immobilized on the working electrode; then, the target biomarker is recognized by the secondary antibody (Ab_2_) labelled with an electroactive species; the target biomarker concentration can be quantified by measuring the electrode current when a potential is applied [29].

Wu et al. developed an electrochemical sandwich ELISA immunosensor based on amino group functionalized silicoaluminophosphate molecular sieves (NH_2_-SAPO-34) supported by Pd/Co nanoparticles, for nuclear matrix protein 22 (NMP-22) detection (Figure 2b) [33]. The large surface area of reduced graphene oxide-NH (rGO-NH), which is used to modify the glassy carbon electrode (GCE), and NH_2_-SAPO-34, which works as the electroactive material, are aimed at improving the number of immobilizing Ab_1_ for NMP22 recognition and the Pd/Co nanoparticles are for the catalysis of the electrochemical reaction, respectively. To eliminate the nonspecific sites on the working electrode, it was incubated in a bovine serum albumin (BSA) solution. The current response linearly correlated with the concentration of the NMP-22, the LOD was 0.33 pg/mL, and the detection range was from 0.001 to 20 ng/mL (Figure 2c). The amperometric response remains virtually unchanged in the presence of interfering substances, suggesting the selectivity of the sensor is acceptable (Figure 2d). Different from BSA occupation, the surface contamination of working electrodes can be avoided by separating biomarker recognition and electrochemical signal generation. Song et al. utilized magnetic nanoparticles (MPs) with Ab_1_ to recognize the matrix metalloproteinase-9 (MMP-9) firstly, then the MPs were transferred to the surface of a tin oxide electrode, which was the working electrode, by applying a magnet on the back of the ITO for further signal generation [34]. Thus, the direct contact between the urine and the working electrode could be avoided in this method and nonspecific absorption could be eliminated.

Except for the sandwich-based model, Giannetto et al. first addressed the competitive approaches for the amperometric immunosensor, which achieved not only the linear range of wild-type p53 from 20 pM to 10 nM, but a low LOD of 14 pM in the synthetic urine samples [35]. After the screen-printed carbon electrodes, which are modified by gold nanoparticles and carbon nanotubes (CNT/GNP SPCE), are directly functionalized with p53 and blocked by BSA, they are incubated with anti-p53 antibodies and alkaline phosphatase-conjugated reading antibodies (RAM-AP) in sequence; then, the amperometric signal can be read (Figure 2e).

##### Label-Free Electrochemical Sensor

**Figure 3:**
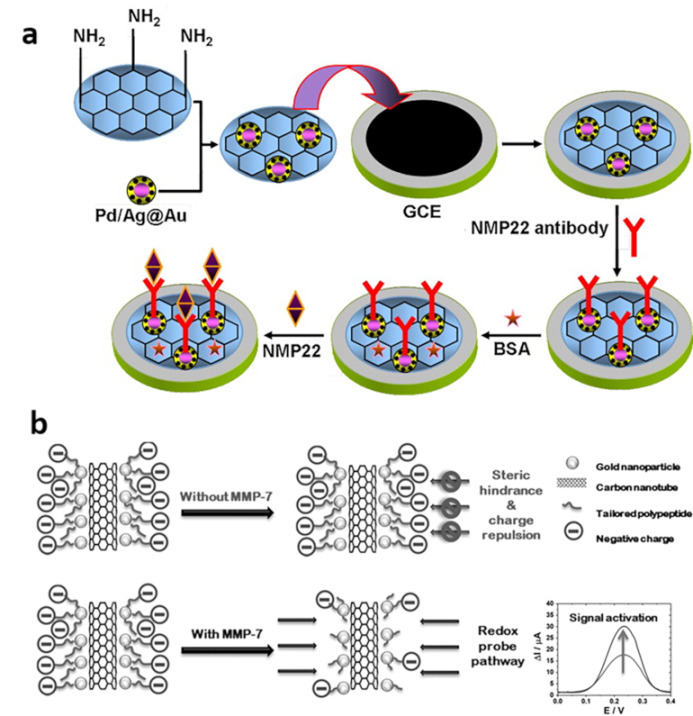
Schematic of the label-free electrochemical sensor. (**a**) The fabrication of the Au@Pd/Ag/NH_2_-GS electrochemical immunosensor. (Reprinted with permission Ref. [38]. Copyright © 2014 Elsevier B.V.) (**b**) Schematic of the operating principle of the sensor reported by Palomer et al. (Reprinted with permission Ref. [40]. Copyright © The Author(s). Published by Elsevier B.V.).

In contrast with eELISA, the label-free electrochemical sensors have the advantages of lower cost and simple fabrication because the use of Ab_2_ is absent [36,37,38]. Li et al. developed a label-free electrochemical immunosensor applying an Au@Pd/Ag/NH_2_-Graphene Sheet (GS) to detect the NMP-22 (Figure 3a) [38]. The concentration of NMP-22 is accessed based on the change of the peak current of K_3_ [Fe(CN)_6_] after the biomarkers are recognized by the antibodies immobilized on the Au@Pd/Ag/NH_2_-GS/GCE. Except for the antibodies, molecularly imprinted polymers (MIP) can also be used in the label-free sensor. Lee et al. applied molecularly imprinted poly (ethylene-co-vinyl alcohol) coated zinc oxide nanorods to develop an NMP-22 detection sensor [39]. The biomarkers, which are enzymes, can enzymatically cleave the specific peptides immobilized on the surface of the working electrode; the electron transfer facilitated by the cleavage reaction can be detected [40]. Based on this principle, Palomar et al. developed a label-free electrochemical sensor for the detection of matrix metalloproteinase-7 (MMP-7) (Figure 3b) [40]. The peptides conjugated to the surface of gold nanoparticles (GNPs)/carbon nanotube (CNT)/gold electrode through gold–thiol bonds inhibit the electrochemical signals for the steric hindrance and charge repulsion and their cleavage results in signal activation.

**Table 2:** Summary of the amperometric sensor reported for bladder cancer urine biomarker detection.

No.	Technique	Biomarker	Working Electrode Modification	Electroactive Species	LOD	Linear Range	Ref.
1	eELISA (sandwich)	NMP-22	Ab_1_/NH_2_-rGO/GCE	Ab_2_-Pd/Co NPs	0.33 pg/mL	0.001–20 ng/mL	Wu et al. (2016) [33]
2	eELISA (sandwich)	MMP-9	Ab_1_-MPs/ITO	Ab_2_-ALP	1.9 pM (standard solution); BRREAK 2.3 pM (standard urine)	10 pM–10 nM	Song et al. (2020) [34]
3	eELISA (sandwich)	NUMA1, CFHR1	Ab_1_/FNAB/gold electrode	Ab_2_-ALP	1.13 ng/mL, BRREAK 0.97 ng/mL	1–100 ng/mL	Arya and Estrela (2018) [41]
4	eELISA (sandwich)	ApoA1	Ab_1_-biotin/avidin/ITO	Ab_2_-ALP	1 pM	1 pM–100 nM	Kim et al. (2019) [42]
5	eELISA (competitive)	p53	CNT/GNP-SPCEs	Anti-p53 Ab/RAM-ALP	14 pM	20 pM–10 nM	Giannetto et al. (2017) [35]
6	Label-free electrochemical sensor	NMP-22	Au@Ab/Pd/Ag/NH_2_-GS/GCE	Au@Pd/Ag NPs	3.3 pg/mL	0.01–18 ng/mL	Li et al. (2014) [38]
7	Label-free electrochemical sensor	NMP-22	MIPs/ZnO nanorods		1.0 pg/mL	128–588 ng/mL	Lee et al. (2016) [39]
8	Label-free electrochemical sensor	NMP-22	Ab/AuPdPt NPs	GCE/rGO-TEPA	0.01 U/mL	0.040–20 U/mL	Ma et al. (2015) [43]
9	Label-free electrochemical sensor	MMP-7	Au electrode/CNT/GNPs- designed peptide substrate	CNT/GNPs	6 pg/mL	1 × 10^−2^ to 1 × 10^3^ ng/mL	Palomar et al. (2020) [40]

Note: (Nuclear mitotic apparatus protein 1, NUMA1; Complement factor H-related 1, CFHR1; 1-fluoro-2-nitro-4-azidobenzene, FNAB; Alkaline Phosphatase, ALP; tetraethylene pentamine, TEPA).

##### Field-Effect Transistor (FET)

**Figure 4:**
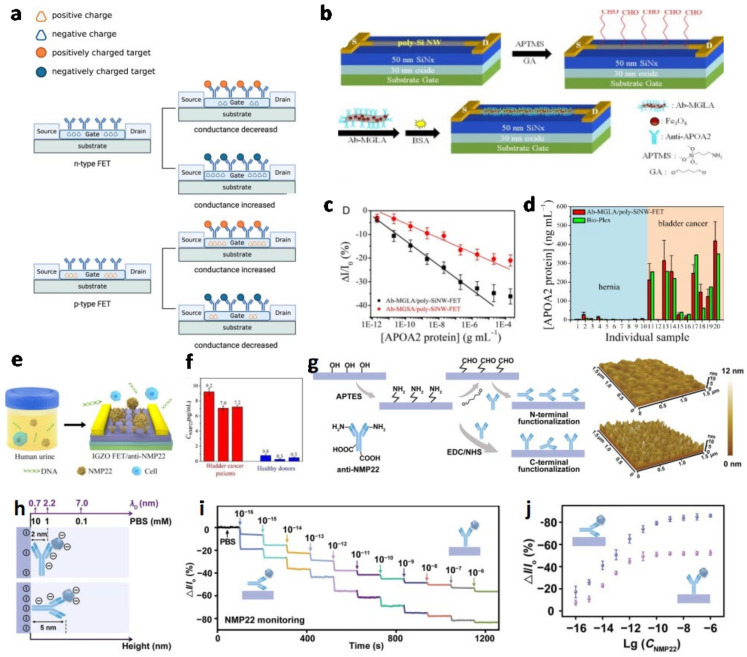
(**a**) The principle of the bio-FET for target biomarker detection (Created with biorender.com). (**b**) The schematic of the preparation of Ab-MGLA/poly-SiNW-FET biosensor reported by Chen et al. (**c**) The calibration curves of the current responses of the poly-SiNW-FET biosensor modified by MGLA and MGSA. (**d**) APOA2 protein concentrations in hernia and BC patients’ urine samples based on Bio-Plex (green bars) and Ab-MGLA/poly-SiNW-FET (red bars). (Reprinted with permission Ref. [22]. Copyright © 2014 Elsevier B.V.) (**e**) Schematic of the BC biomarkers detection with IGZO-FET biosensor. (**f**) Sensing performance evaluation of anti-NMP22/IGZO-FET biosensors towards BC patients’ and healthy donors’ urine samples. (Reprinted with permission Ref. [48]. Copyright © 2020 Elsevier B.V. on behalf of the Chinese Chemical Society and Institute of Materia Medica, Chinese Academy of Medical Sciences.) (**g**) Schematic illustration and AFM images of anti-NMP22 antibodies modification by C-terminal (**bottom**) and N-terminal (**top**) on the IGZO device. (**h**) Debye lengths of protein height and PBS buffer. (**i**) Real-time current response of C-terminal and N-terminal modified anti-NMP22/IGZO FET biosensors. (**j**) Calibrated curves of C-terminal and N-terminal modified IGZO FET biosensors. (Reprinted with permission Ref. [49]. Copyright © 2022 Wiley-VCH GmbH).

Field-effect transistor biosensor (Bio-FET), which can be used in the biomarker test, is an integration of a bio-receptor (such as antibodies, aptamers, peptides, etc.) and an ion-sensitive field-effect transistor (ISFET). The capture of the biomarker by the bio-receptor immobilized on the sensing channel, which is the connection of the source (S) and drain (D) electrodes, affects channel conductivity. For an n-type Bio-FET, the conductivity can be increased or decreased by the accumulation or depletion of electrons as the charge carriers when the positively or negatively charged biomarkers are recognized by the bio-receptor, respectively. For a p-type Bio-FET, where the holes are charge carriers, the channel conductivity can be changed and vice versa (Figure 4a) [44]. A Bio-FET constructed from a nanomaterials-based channel has a more improved performance, which can be applied to achieve a biomarker test with superior sensitivity and an ultra-fast response [45]. In Table 3, the parameters of Bio-FETs for BCa urine cancer biomarker detection are summarized.

Chen et al. first applied FET to detect the bladder cancer biomarker apolipoprotein A-II protein (APOA2). The channel is made of an n-type polycrystalline silicon nanowire (poly-SiNW), which is modified by magnetic graphene conjugated with long-chain acid groups (MGLA) (Figure 4b) [22]. The long-chain acid groups increase the quantity of immobilized anti-APOA2 (Ab) antibodies to give them an increased surface area compared with short-chain acid groups, and preserve the bioactivity of the antibodies for their magnetic properties to enable magnification via the in situ synthesis of the Fe_3_O_4_ NPs on the surface. The LOD of the Ab-MGLA/poly-SiNW-FET is 6.7 pg/mL, and it shows a linear relationship between the relative response and the logarithmical concentration in a range from 19.5 pg/mL to 1.95 µg/mL (Figure 4c). It can also be applied to distinguish the APOA2 concentrations in bladder cancer and hernia patients (Figure 4d). The results indicate that the Ab-MGLA/poly-SiNW-FET is accurate and feasible [22]. Yang et al. utilized the molybdenum disulfide (MoS_2_) nanosheet as the channel for its electronic properties [46]. The biomarkers, NMP-22 and cytokeratin 8 (CK8), could be simultaneously recognized by their antibodies, which are conjugated on the different MoS_2_ sensing channels in an FET sensor array. The MoS_2_ FET biosensor has a linear detection range from 10^−6^ to 10^−1^ pg/mL of NMP22 and CK8, and their LOD are 0.027 and 0.019 aM, respectively. Additionally, not only was its response current for the target protein high, while that of the non-target protein was extremely low, but it could effectively differentiate the urine sample of patients from that of healthy donors [46]. Except for the antibodies, the single-stranded DNA was used as the bio-receptor in the Bio-FET by Guo et al. to detect the microRNA-21, which is also a bladder cancer biomarker [47].

In 2020, Li et al. constructed an indium gallium zinc oxide field transistor (IGZO-FET) with high stability for NMP22 identification in urine samples (Figure 4e) [48]. Except for the extremely low LOD (2.7 amol/L) toward the biomarker being realized, the direct detection of urine biomarkers with high sensitivity and specificity was also achieved. The high-performance biosensor could make a distinction between the urine sample of bladder cancer patients and those of healthy donors (Figure 4f). Although high sensitivity and specificity have been accomplished in various publications, there is still a problem that has not been generally discussed in the area of biomarker detection from urine samples. The problem is that the extremely high ionic strength of urine samples means the surface charge of target proteins need to be screened from the surrounding charged ions through Debye screening, which decreases the effective charge of the protein and then upsets the identification. Based on this problem and previous research, Yang et al. regulated the arrangements of the antibodies on the IGZO surface of the IGZO-FET to minimize the occupied length and reduce the distance between target protein biomarkers and the IGZO channel (Figure 4g) [49]. They compared the performance of the N-terminal functionalized anti-NMP22 IGZO-FET biosensor and the N-terminal functionalized biosensor, which revealed that the N-terminal strategy efficiently minimized the occupied length (Figure 4h), yielded a higher current response toward NMP22 (Figure 4i), and boarded the linear detection range in the 10 × 10^−3^ M PBS, which had the equivalent ionic strength to the urine (Figure 4j). In addition, a bladder cancer identification accuracy of 95.0% and a cancer stages classification accuracy of 90.0% was attained through the integration of a machine-learning algorithm.

**Table 3:** Summary of the field-effect transistor-based sensor for bladder cancer urine biomarker detection.

No.	Biomarker	Gate	LOD	Linear Range	Ref.
1	APOA2	Ab-MGLA/poly-SiNW	6.7 pg/mL	9.5 pg/mL and 1.95 µg/mL	Chen et al. (2015) [22]
2	NMP22, CK8	Ab-MoS_2_	0.027 aM, 0.019 aM	10^−6^–10^−1^ pg/mL	Yang et al. (2020) [46]
3	microRNA-21	ssDNA-IGZO	19.8 amol/L	---	Guo et al. (2021) [47]
4	NMP22	Ab-IGZO	2.7 amol/L	0.0001–0.1 pg/mL	Li et al. (2020) [48]
6	NMP22	N-terminal Ab-IGZO	3.2 × 10^−17^ g/mL	10^−16^–10^−12^ g mL^−1^	Yang et al. (2022) [49]
5	APOA1	Ab-SiNW	1 ng/mL	0.2 ng/mL-10 μg/mL	Lin et al. (2017) [50]

Note: (IGZO is short for indium gallium zinc oxide).

#### 2.1.2. Optical Methods

Optical techniques have the advantages of not requiring a washing procedure, rapid detection, the signal can be identified by the naked eye when used for biomarker detection, and they show potential for exploitation in POC situations for early cancer diagnostic [51].

##### Fluorescence Assays

**Figure 5:**
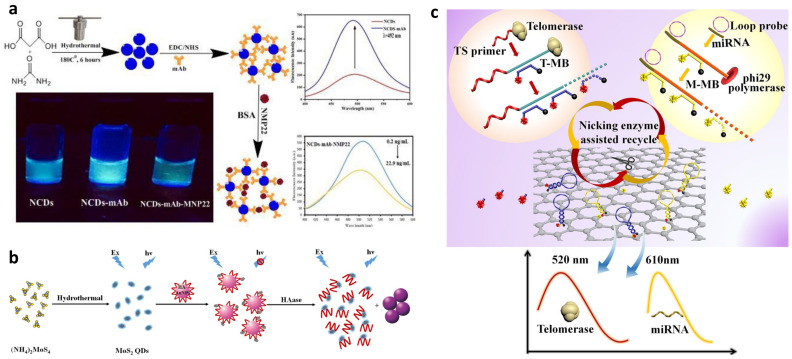
(**a**) Schematic for the detection principle of NMP22 with carbon dots. (Reprinted with permission Ref. [55]. Copyright © 2020 Elsevier B.V.) (**b**) Schematic of the synthesis of MoS_2_ QDs and HAase detection. (Reprinted with permission Ref. [56]. Copyright © 2016 American Chemical Society.) (**c**) Schematic of the binary assay for miRNA and telomerase. (Reprinted with permission Ref. [59]. *Mater. Interfaces* 2017, 9, 28, 23420–23427. Copyright © 2017 American Chemical Society).

Since two *Science* articles established quantum dots (QDs) as a new kind of fluorophores and indicated their advantages in 1998 [52,53], they have been widely applied in the field of biomolecule sensing and imaging [27,54]. The QDs-based fluorescence assay developed for urine biomarker sensing follows a similar principle, which is either that the quantum dots’ fluorescence is quenched in the presence of the biomarker or it is quenched in the absence of the biomarker by a quencher and recovered when the quencher is reacted in the presence of a biomarker. Othman et al. synthesized monoclonal antibody-conjugated nitrogen-doped carbon quantum dots (mAb-NCDs) for the fluorescence immunoassay of NMP22 (Figure 5a) [55]. The fluorescence quenching caused by the formation of the immunocomplex on the carboxylated NCDs and the decrease in fluorescence intensity is proportional to the concentration of NMP22 in a linear range of 1.3–16.3 ng/mL and an LOD of 0.047 ng/mL. Gu et al. applied MoS_2_ QDs and hyaluronic acid-functionalized gold nanoparticles (HA-AuNPs) to develop a fluorescence assay for the detection of hyaluronidase (HAase) in urine [56]. In the absence of HAase, the fluorescence of MoS_2_ QDs is quenched by HA-AuNPs by photoinduced electron transfer (PET) in the nano assembly of MoS_2_ QDs/HA-AuNPs. When the HAase presents, the disassembly occurs and the AuNPs aggregate as a consequence of HA cleavage (Figure 5b). Then, the fluorescence of MoS_2_ QDs recovered. The linear range of the assay is from 1 to 50 U/mL and the LOD is 0.7 U/mL. Except for the urine biomarker test, Xu et al. also utilized core-shell-shell magnetic quantum dot microbeads in serum microRNA detection [57].

Because the urinary level of telomerase is highly related to bladder cancer, it has been regarded as a promising biomarker for bladder cancer diagnosis [58]. Duan et al. developed a binary fluorescence assay that can simultaneously detect telomerase and microRNA-21 (miR-21) based on the telomerase extension reaction and miR-21 microRNA-induced rolling circle amplification (Figure 5c) [59]. Graphene oxide is applied to decrease the background noise and a nicking enzyme is used to enhance the fluorescence signal. The LOD for telomerase and miR-21 are 0.4 cells/µL and 1 pM, respectively. Additionally, the binary strategy also shows the potential to differentiate between MIBC and NMIBC, which can contribute to its clinical application.

Detection accuracy and sensitivity can be further improved by applying the ratio of two different emission intensities in the ratiometric fluorescence assay [60]. Raj et al. reported a ratiometric fluorescence assay for the detection of HAase based on the Förster resonance energy transfer (FRET) mechanism [61]. The positively charged naphthalimide and cationic carbon dots are absorbed by negatively charged hyaluronic acid and the supramolecular nano assembly is formed. A FRET emission of 542 nm is obtained from carbon dots to naphthalimide when the assembly is in an aggregate state. In the presence of HAase, the assembly changed into a dispersive state for the cleavage of hyaluronic acid; the FRET emission was disrupted and an emission of 416 nm was obtained. The concentration of HAase was correlated with the intensity ratio of 416 nm, a 542 nm emission, and a LOD of 0.09 U/mL was obtained. Ma et al. also developed a ratiometric fluorescent probe based on QDs for the detection of telomerase [62].

Autofluorescence emitted from impurities in a complex urine sample matrix causes high background noise and prevents improvement in the sensitivity of biomarker detection [63]. To solve this problem, long-lifetime luminescence can be exploited for it can be detectable when the autofluorescence decays rapidly after the excitation is removed [63]. Wang et al. developed an optical biochip based on photonic crystals (PCs)-supported long-lifetime luminescence nanoparticles (LLNPs), which were functionalized by single strand DNAs (denoted as cDNAs) for the detection of bladder cancer-related miR-21 through hybridization [63]. In the absence of miR-21, the luminescence can be quenched by black-hole-quencher-labelled DNAs (BHQ-DNAs), which are partially hybridized with cDNA through a FRET mechanism from LLNPs to BHQ dyes. The miR-21 more competitively hybridized with cDNAs and led to the detachment of the BHQ-DNAs, and then, the long-lifetime luminescence was consequently returned. In addition, PCs, which can reflect light within a certain wavelength range, were utilized to enhance the signal and a LOD of 26.3 fM was reached.

##### Dynamic Light Scattering

**Figure 6:**
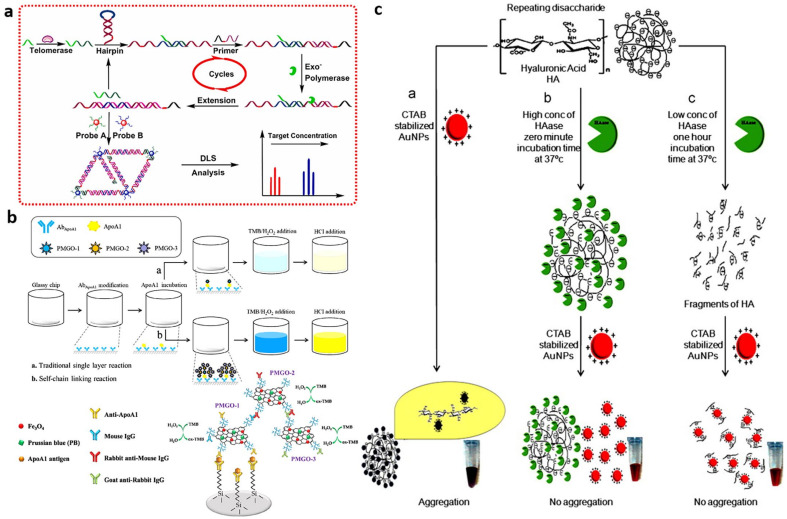
(**a**) Schematic of the telomerase detection strategy. (Reprinted with permission Ref. [58]. Copyright © 2019 Elsevier B.V.) (**b**) Schematic of the traditional signal (**up**) and self-chain linking reaction (**down**) for the ApoA1 detection. (Reprinted with permission Ref. [66]. Copyright © 2018 Elsevier B.V.) (**c**) The mechanism of cationic AuNP-based colorimetric assay for the direct detection of HAase activity. (Reprinted with permission Ref. [67]. Copyright © 2013 Elsevier B.V.).

The telomerase substrate (TS) primer can be elongated with the (TTAGGG)_n_ repeating sequences in the presence of telomerase, producing the telomerase extension product (TEP). Wang et al. applied TEP to trigger the strand displacement amplification (SDA) between the primer and the hairpin deoxyribonucleic acid (DNA) and the resulting product was detected by oligonucleotide-modified AuNP probes (Figure 6a) [58]. By measuring the diameter of the AuNPs’ aggregation with dynamic light scattering (DLS), the number of cancer cells was known and an LOD of three MCF-7 cells was achieved. Except for the SDA process, the hybridization chain reaction (HCR) [64] and catalytic hairpin assembly (CHA) [65] has also been exploited in AuNPs-based-DLS detection, and an LOD of 4 or 10 MCF-7 cells was achieved, respectively.

##### Colorimetric Assay

To overcome the disadvantage of the instability of horseradish peroxidase (HRP), which is used in the conventional ELISA, Peng et al. utilized stable, self-linkable Prussian blue (PB)-incorporated magnetic graphene oxide (PMGO) to develop an ultrasensitive colorimetric immunosensor, which can detect the concentration of ApoA1 in urine (Figure 6b) [66]. After the ApoA1 proteins were captured by the ApoA1 antibody-functionalized chip, they were also incubated with the PMGO functionalized by the ApoA1 antibody and mouse IgG (PMGO-1), rabbit anti-mouse IgG antibody (PMGO-2), and goat anti-rabbit IgG antibody (PMGO-3), simultaneously. As a peroxidase-mimicking nanozyme, the PMGO oxidizes 3,3′,5,5′-tetramethylbenzidine (TMB) and then an amplified absorption intensity is generated. The linear detection range of 0.05 ng/mL to 100 ng/mL was achieved, which is wider compared with that without signal amplification.

For the visible colour shift of AuNP colloidal solution from red (dispersion) to blue (aggregation) due to the surface plasmon resonance (SPR) phenomenon, AuNPs can be exploited to develop rapid and simple colorimetric assays for urine bladder cancer biomarker detection [23,67]. Based on the unique optical property of AuNPs, Nossier et al. utilized the cetyl trimethyl ammonium bromide (CTAB) stabilized AuNPs and HA to develop a direct colorimetric assay for the detection of HAase in urine, and its concentration was reflected by the unchanged red colour of the AuNPs (Figure 6c) [67]. Compared with the zymography method, this strategy showed better sensitivity (82.5% vs. 65%) and comparable specificity (96.1% vs. 96.1%) [67]. A similar principle has also been applied for gelatinase detection [68].

The aggregation-dispersion method can also be combined with Fenton’s reaction for the construction of a colorimetric biomarker assay. Acharya et al. introduced a clinical assay for matrix metalloproteinases (“Ammps”) in this manner [69]. The assay substrates (aggregation) were generated by crosslinking the Fe (II) chelated alginate nanoparticles via gelatin, and the free-floating nanoparticles (dispersion) were produced via cleavage due to the presence of collagenases in the urine sample. After the removal of alginic acid chelate by adding acid and the activation of the Fe (II) catalyst, a visual signal was obtained for the colour generation of the Fenton’s reaction. As a POC urine test, the Ammps had a sensitivity of 100% and a specificity of 85% in the pilot study of 88 patients [69].

### 2.2. Urinary Nano-Cytology

**Figure 7:**
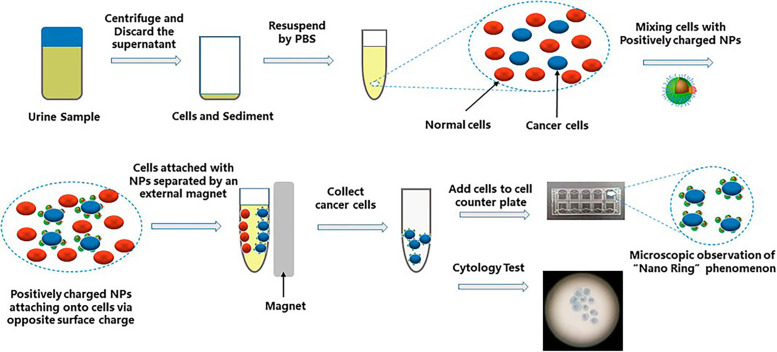
Schematic of nano-cytology. (Reprinted with permission Ref. [71]. Copyright © 2022 Xu, Zeng, Li, Gao, Le, Huang, Wang, Chen, Zhang and Xu.).

Although lots of urine bladder cancer biomarker tests have been invented, urine cytology is still the only recommended method for the surveillance of bladder cancer under different guidelines [70]. To improve the sensitivity of cytology, Xu et al. mixed the positively charged multifunctional nanoprobes (Fe_3_O_4_@SiO_2_, mNPs) with urine to identify the cancer cells (Figure 7) [71]. A blinded and single-centre clinical trial was performed with urine samples from 102 patients; considering the comparable specificity, simultaneously, nano-cytology may be a new promising assay for the diagnosis of BC. Bladder cancer patients and 49 non-cancer participants were used to compare the sensitivity of the nano-cytology and conventional cytology. The results showed a significantly increased overall sensitivity, which was 82.4% in the nano-cytology group compared with the conventional cytology group, whose sensitivity was 58.8% [71].

### 2.3. Nanomedicine Advanced Imaging Techniques in the Bladder Cancer Diagnosis

#### 2.3.1. Optical Imaging in Bladder Cancer Diagnosis

##### In Vivo Imaging in Bladder Cancer Diagnosis

Nowadays, the widest applied method for bladder cancer diagnosis is white light cystoscopy (WLC), which is based on the colour and texture images of superficial tissue changes [23,72]. The transurethral resection (TUR), guided by WLC after a suspicious lesion is identified, is the standard care for non-muscle invasive bladder cancer (NMIBC) [73]. However, aside from the discomfort and the risk of infection from the invasive method, its inability to detect carcinoma in situ (Tis) and some small or satellite tumors has become a challenge.

**Figure 8:**
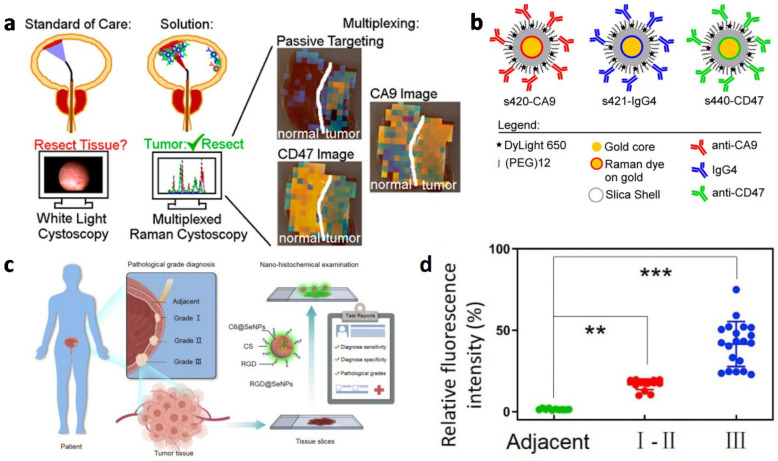
(**a**) Schematic of surface-enhanced Raman scattering nanoparticles (NPs) for the multiplexed imaging of BC tissue. (**b**) Schematic of SERS nanoparticles. (Reprinted with permission Ref. [73]. Copyright © 2018 American Chemical Society.) (**c**) Schematic illustration of the RGD polypeptide modified cancer-targeted selenium nano-system (RGD@SeNPs) for advanced nanohistochemical examination. (**d**) Statistical analysis of the diagnostic results with 100 μM RGD@SeNPs nanoparticles, *p* < 0.01 (**) or *p* < 0.001 (***) (scale bar = 200 μm). (Reprinted with permission Ref. [86]. Copyright © 2021, Elsevier Ltd.).

Molecular imaging can be utilized to improve the white light-based method. Davis et al. designed a surface-enhanced Raman Scattering (SERS) nanoparticle-endoscope system as a supplement to WLC, and multiplex molecular imaging is achieved for the sharp spectral lines (<1 nm) produced by the SERS nanoparticles [73]. After the intravesical SERS nanoparticles are administered, both tissue permeability-based (passive) and antibody-based (active) targeting mechanisms are evaluated (Figure 8a,b). The ex vivo experiments presented a receiver operating characteristic area under the curve (ROC AUC) of 0.93 (0.73, 1.0) and 0.93 (0.75, 1.0) for the 1-plexed passively targeted nanoparticle imaging and 3-plexed CD47, CA9, and passively targeted nanoparticles imaging, respectively. It is indicated that the method reported can improve the residual disease identification ability during WLC. In addition, the tissue penetration depth was 5-fold higher in tumors than that of normal tissues on average; it is suggested that it has an enhanced surface permeability and retention (ESPR) effect in bladder cancer. Furthermore, the 3-plexed active-to-sum method is more accurate than the 2-plexed active-to-sum method, indicating that multiplexed molecular detection with Raman endoscopy could boost identification. Generally, the results of the paper supported that intravesical SERS nanoparticles-based Raman endoscopy is a potential method for NMIBC resection improvement.

For the detection of NMIBC, hexaminolevulinate fluorescence cystoscopy, also known as blue light cystoscopy (BLC), and photodynamic diagnosis were developed [74,75]. After the photosensitizers, such as 5-aminolevulinic acid (ALA) or its hexyl ester, hexaminolevulinate 5-ALA (Hexvix^®^ [HAL], Photocure, Oslo, Norway) are instilled into the bladder, the tumor tissue absorbs them more than normal tissue. For ALA, the ratio of the quantity that accumulates within the tumor and normal tissue is 20:1. Under the irradiation of blue light, red light is emitted and detected, thus, the tumor appears red compared with the normal tissue [75]. Studies supposed that the uptake of ALA is similar in all cell types and its accumulation in tumor tissue is due to the differences in the conversion and elimination process [74]. For the increased uptake of nanoparticles in tumor tissues, nanomaterials-based photosensitizers can be applied to overcome the shortcoming of hexaminolevulinate’s low specificity, especially after intravesical Bacillus Calmette-Guérin (BCG) therapy [76]. Lin et al. coated the nanoporphyrins with a bladder cancer-specific ligand, PLZ4, to construct the photosensitizers [77]. Under the illumination of near-infrared light, the PLZ4-nanoporphyrin can emit fluorescence, heat, and reactive oxygen species, allowing for efficient and specific detection. Pan et al. developed a method where fluorescent quantum dots were attached to anti-CD47 antibodies that are instilled into the bladder; then, the bladder regions are analyzed via blue light cystoscopy. This was used in a variety of bladder cancers, including Tis, residual carcinomas in tumor resection beds, recurrent carcinomas following prior BCG therapy, and excluded cancers from benign but suspiciously-appearing mucosa. The 199 bladder regions were analyzed and a sensitivity of 82.9% and a specificity of 90.5% were achieved.

In recent years, tumor-related mRNAs have been widely exploited, as cancer biomarkers, for cancer diagnosis development [78,79,80]. The novel spherical nucleic acids (SNAs) nanoparticle conjugates have been utilized for mRNA monitoring and these nanoprobes first enter cells through endocytosis and then are released into the cytosol [81]. However, the abundance of lysosomal enzymes tends to destroy the functional nucleic acids, which may significantly decrease detection efficiency [82]. Ma et al. found that a poly(11)-arginine cell-penetrating peptide termed R11, which exhibited the highest uptake efficiency with regard to bladder cancer cells, can promote SNAs to escape from lysosomes and then enter the cytoplasm so that the SNAs can successfully achieve hybridization with the target and the rapid release of the fluorescence [83]. They modified the gold nanoparticles-molecular beacon (AuNP-MB) with R11 to detect survivin mRNA, which is abundant in bladder cancer and present in early-stage bladder cancer. AuNP-MB@R11 showed high sensitivity and specificity, and also exhibited good stability against nucleases in vitro. Furthermore, AuNP-MB@R11 can accumulate in bladder cancer tissues after intravesical injection without causing injury to other organs. To sum up, the AuNP-MB@R11 is expected to be an intraoperative molecular imaging technique that can achieve an accurate determining and complete resection of bladder tumors, further improving patient outcomes.

##### Biopsy in Bladder Cancer Diagnosis

For the tumor histopathological diagnosis, tissue biopsy is still the fundamental choice as the final clinical diagnostic pathology [84]. To remove the obstruction of widely applied conventional immunohistochemical (IHC) technology, which is costs time and is relatively expensive due to the use of antibodies [85], Liu et al. designed an RGD polypeptide modified cancer-targeted selenium nano-system (RGD@SeNPs) for advanced nanohistochemical examination, which has the advantages of not only rapid visualization but the pathological grading of bladder cancer tissues (Figure 8c) [86]. This RGD@SeNPs-based strategy has a specificity of 98% and a sensitivity of 92% when dealing with the differentiation between bladder tumor tissues and normal tissues via the examination of bladder tumor specimens and non-tumor specimens. Moreover, based on the statistical results of fluorescence intensity, the stage grading can be realized and it is consistent with the clinical diagnosis (Figure 8d).

#### 2.3.2. Magnetic Resonance Imaging in Bladder Cancer Diagnosis

Magnetic resonance imaging (MRI) techniques visualize the specific atomic nuclei spins within the body based on chemical nuclear magnetic resonance (NMR) analysis [87]. Nowadays, MRI is one of the widest-used techniques for evaluating tumor staging in bladder cancer in the clinic [23]. Nanomaterials-based contrast agents are a useful approach to improving MRI performance. Sweeney et al. engineered the bladder cancer-specific peptide cyc6-functionalized mesoporous silica nanoparticles (MSNs), which were incorporated by the MRI contrast agent Gd_2_O_3_ to visualize tumor boundaries in the T1- and T2-weighted MRI [88]. The method has an improved specificity due to the improved bladder cancer cells affinity of the particles via the molecular target. Unlike monoclonal antibodies, the cyc6 peptide has the advantage of being non-immunogenic, which gives this research the potential of being translated.

Except for local tumor detection, lymph node evaluation is also important, as the extent of metastatic lymph nodes is also under consideration when assessing treatment and prognosis [89]. Conventionally, node detection relies on their size and shape; however, enlarged, reactive, non-malignant lymph nodes may be misdiagnosed as metastatic lymph nodes. Thus, techniques that can achieve nodal function evaluation rather than morphology have been invented. Deserno et al. used an ultrasmall superparamagnetic iron oxide (ferumoxtran-10) particle-enhanced MRI to detect node metastases smaller than 10 mm [89]. Because of the uptake of ferumoxtran-10 into normal nodal tissue and a lack of uptake for metastases infiltrated nodal areas, the signal intensity of T2- or T2*-weighed MR images for normal nodal tissue is decreased while that of metastases infiltrated nodal areas is retained.

#### 2.3.3. Multimodal Imaging

Although current imaging modalities play an important role in tumor diagnosis, there is no single-modality imaging technique that can satisfy the spatial resolution, signal sensitivity, and tissue penetration depth required for complete imaging. To solve this problem, multimodal imaging can be achieved by the integration of multi-imaging models into a single nano-system to obtain more imaging information and to help make a more accurate diagnosis. Key et al. engineered multimodal imaging cyanine 5.5 fluorescence molecules with modified-chitosan nanoparticles, which capture the ferrimagnetic nanocubes (NCs) for both magnetic resonance imaging and near-infrared (NIR) fluorescence [90]. The ability of the multimodal nanoparticles to visualize small tumors is also confirmed.

## 3. Nanomedicine-Based Bladder Cancer Therapy

### 3.1. Nanocarriers-Based Intravesical Delivery for Chemotherapy of Bladder Cancer

As the standard therapeutic schedule for bladder cancer patients, surgical transurethral resection may incompletely resect, or miss, small malignant lesions that have the potential to lead to recurrence [91]. To avoid recurrence and progression after surgery, intravesical instilled chemotherapy and immunotherapy allow cancer lesions to be directly exposed to therapeutic agents, and are widely accepted as adjuvant therapies [92]. However, there are still lots of problems with conventional intravesical chemotherapy and immunotherapy. The deficiencies in the therapeutic efficacy of intravesical instilling chemotherapy drugs, such as the weak penetration ability of the urothelium and the short residence time of the therapeutic agents in the bladder, have already been indicated [15]. The uroplakins and transmembrane proteins consist of covering plaques, tight cell junctions, and the glycosaminoglycan mucin layer of the mucosal layer, prevent the therapeutical agents from effectively penetrating into tumors [93,94,95]. Additionally, the filling of the bladder causes an urge to urinate and the concentration of the therapeutic agent can be diluted by urine [92]. To maintain the concentration, frequent catheterizations are necessary but these lead to bladder irritation, infection, fibrosis and inflammation [11]. Thus, future improvements to overcome these disadvantages are needed. Nowadays, advances in nanomedicine and biomaterials have indicated a high-profile and promising pathway to deliver anticancer agents and improve current therapies. Moreover, many clinical trials of nanostructure-based chemotherapy in bladder cancer have been conducted [92]. This section will focus on the nanomedicine-based novel drug delivery system for intravesical chemotherapy.

#### 3.1.1. Mucoadhesive Nanocarriers for Intravesical Drug Delivery

For the formation of hydrogen bonds between the mucin layer and the hydrophilic mucoadhesive materials, an intravesical drug delivery system can be utilized to increase the dwell-time and penetration of the therapeutic agents carried, i.e., they can attach to the mucous membrane of the urothelium, which enables the formulation to be maintained for a longer period and ensures an intensive contact with the mucous layer [11]. The carrier should be rapidly attached to the bladder wall after instillation and the formulation must not interfere with the normal functions of the bladder. In addition, the attachment or adhesion should be retained even after the voiding of urine [93]. The mucoadhesive properties of many biomolecules and synthetic polymers have been identified and they can be used as mucoadhesive carriers for intravesical drug delivery [11]. Despite the attachment efficacy of the delivery nanocarrier, the biosafety of the whole therapeutic system is another important factor that determines its practical application in the clinic.

##### Synthetic Polymeric Mucoadhesive Nanocarriers

The variety of compositions of polymer-based nanoparticles provides the advantage of being able to load a variable amount of a drug. It should be noted that only biocompatible and biodegradable synthetic polymers can be used for drug delivery. The loaded drug’s activity should be maintained, adverse interactions between the drug and the polymer should be avoided, the delivery system should have sufficient shelf-time, and optimal drug exposure should be allowed [11].

As a biodegradable, biocompatible, and mucoadhesive polymer [96], poly(ethyl-2-cyanoacrylate) (PECA) can be a good intravesical drug carrier. Chang et al. investigated PECA epirubicin-loaded nanoparticles (EPI-NP) for intravesical instillation against bladder cancer cell lines (T24, RT4) [97]. The surfactant Tween80 or pluronic was used for the emulsion polymerization of the nanoparticles with sizes less than 200 nm. Compared with the low efficacy of the commercial aqueous formulations of EPI, the EPI-NP formulation showed greatly improved penetration of epirubicin into the bladder wall. There was no adverse structural damage caused to the urothelium by the formulation, which was proved by histological staining.

To substitute human serum albumin (HSA), a dendritic-like hydrophobically derivatized hyperbranched polyglycerol (HPG) with a hydrophilic shell and a hydrophobic core was synthesized [98]. Its transport and binding properties were also investigated. The mixture of the alkyl (C8/C10) chains-based hydrophobic core was essential for the binding of the hydrophobic drug, while the methoxy-poly (ethylene glycol) (MePEG, MW 350) based hydrophilic shell was significant for its good water solubility [99]. Mugabe et al. modified HPGs with amine groups through the derivatization of the outer surface hydroxyl groups, which improved their mucoadhesive properties, and used the HPG-C8/10-MePEG-NH_2_ nanoparticles as the docetaxel (DTX) carrier for the intravesical chemotherapy [100]. The rapid take-up of these nanoparticles into cells was proved, the highly loaded DTX in the HPGs was confirmed, and the continuous controlled release was also characterized. Compared with the commercial formulation of Taxotere, the DTX-loaded HPG-C8/10-MePEG-NH_2_ nanoparticles showed a higher efficacy in reducing the growth of the orthotopic bladder cancer model [100]. The effects of HPG-C8/10-MePEG-NH_2_ nanoparticles on bladder urothelial integrity and morphology were subsequently studied [101]. The significant but rapid recoverable changes in the morphology of the urothelium caused by these nanoparticles suggest that they are suitable for intravesical drug delivery.

Due to the nonspecific interaction between the mucous membrane and poly(ethylene glycol) (PEG) chain, it seems to be one of the most promising mucoadhesive polymers [102]. Guo et al. prepared a nanogel of oligoarginine-poly(ethylene glycol)–poly(L-phenylalanine-co-L-cystine) (R9-PEG–P(LP-co-LC)) to enhance the penetration of a chemotherapy drug toward the bladder wall [103]. The nonspecific interaction between the bladder mucosa and the PEG chain and the electrostatic interaction between the negatively charged mucosa and the cationic cell-penetrating peptide R9 were accompanied to enhance the mucoadhesive capability of the carrier. The 10-hydroxycamptothecin (HCPT)-loaded R9-PEG–P(LP-co-LC) (i.e., R9NG/HCPT) demonstrated a triggered release behavior for the cleavage of the disulfide bond in the intracellular reductive microenvironment, and superior cytotoxicity against bladder cancer cells. The experiments concerning the weight loss and survival periods of HCPT-loaded nanogel-treated rats demonstrated, well, the biological safety of the therapeutic system. The favorable results of both the in vitro experiments against human BC 5627 cells and the in vivo experiments against orthotopic bladder cancer models in mice and rats indicate the potential of this advanced nanoformulation for use in clinic.

##### Chitosan-Based Mucoadhesive Nanocarriers

Chitosan is one of the widest-used polysaccharides for the enhancement of drug permeability through the urothelium [11]. Due to its biocompatible, bioactive, biodegradable and polycationic properties, as well as its reactive amine and alcohol groups, chitosan has advantages for intravesical delivery [104]. In 2002, the application of chitosan carriers to deliver mitomycin C (MMC) for the intravesical chemotherapy of superficial bladder cancer was evaluated by Eroglu and colleagues [104]. The results of the bioadhesion tests, which were performed with sheep bladders, indicated that the chitosan with a larger molecular weight had higher adhesive strength since the larger molecular weight chitosan can interact better with the glycosaminoglycans in the mucous membrane. In addition, the controlled release of MMC was achieved with the chitosan carrier.

In 2009, Bilensoy et al. compared the effect of the cationic nanoparticles of chitosan (CS), poly-ε-caprolactone coated with chitosan (CS-PCL), and poly-ε-caprolactone coated with poly-L-lysine (PLL-PCL), on the MMC carrier for the intravesical chemotherapy of bladder cancer [105]. The CS-PCL nanoparticles can realize complete drug release while the CS and PLL-PCl cannot, due to strong polymer-drug interactions. CS-PCL was the most efficient carrier for the uptake of hydrophobic or hydrophilic fluorescent markers against normal cells and cancer cells. Especially, hydrophilic Rhodamin123 loaded CS-PCL nanoparticles were selectively intergraded by the MB49 bladder cancer cell line while they were not incorporated by the G/G normal bladder epithelial cells. For the shared hydrophilic properties of Rhodamin123 and MMC, the selective delivery ability of the CS-PCL nanoparticles may be utilized in the targeted therapy for superficial bladder cancer. Chitosan nanoparticles can also be utilized for the delivery of other therapeutic agents, such as cisplatin [106], and paclitaxel [107,108].

**Figure 9:**
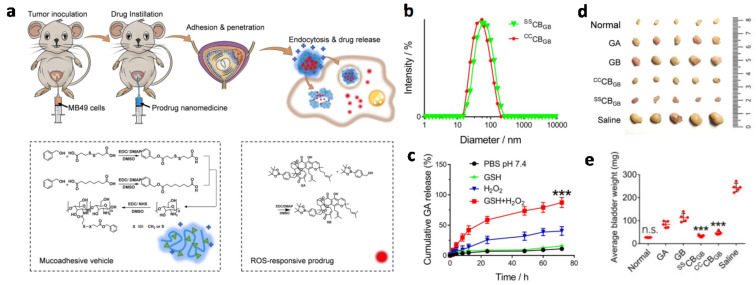
(**a**) The schematic of the intravesical instillation of chitosan-based GSH responsive nanoparticles for prodrug delivery reported by Xu et al. (**b**) Rh of ^SS^CB_GB_ and ^CC^CB_GB_ nanoparticles. (**c**) Release profiles of GA from ^SS^CB_GB_ nanoparticles under different environments. *** *p* < 0.001 for GSH + H_2_O_2_ group compared with the other groups. (**d**) The appearance and (**e**) the average weight of normal bladder and saline, GA, GB, ^SS^CB_GB_ and ^CC^CB_GB_ treated bladder. (Reprinted with permission Ref. [109]. Copyright © 2020 Elsevier B.V.).

To avoid damage to the normal urothelium and the urethra, which is caused by common cytotoxic payloads such as doxorubicin, cisplatin, paclitaxel, mitomycin or gemcitabine, advanced drug delivery systems with high selectivity have been developed. For instance, a positively charged chitosan-based, intracellular glutathione (GSH) responsive nanoparticle has been invented by Xu and colleagues for the delivery of the reactive oxygen species (ROS)-activated gambogic acid prodrug (noted as GB), to achieve a tumor-selective intravesical instillation based on small molecule chemotherapeutics [109]. The drug, Gambogic acid (GA), can only be generated inside bladder cancer cells due to the higher concentration of GSH and ROS; it exhibited a high selectivity and exempted the normal urothelium (Figure 9a). Part of the amino groups of chitosan were conjugated with a hydrophobic group that contains a benzyl group for more efficient encapsulation of the hydrophobic cargos. The 3,3′-dithiodipropionic acid and adipic acid were reacted with benzyl alcohol to generate the GSH responsive and non-responsive intermediates, respectively, and then were conjugated to chitosan to yield responsive (^SS^CB) and non-responsive carriers (^CC^CB) (Figure 9a). The average size of the GB-loaded ^SS^CB and ^CC^CB, (i.e., ^SS^CB_GB_ and ^CC^CB_GB_) were 83.4 nm (PDI = 0.183) (Figure 9b) and 78.8 nm (PDI = 0.207), respectively, and were stable during 7 days incubation in PBS. The high-performance liquid chromatography (HPLC) results indicated an efficient release of GA under an environment of both 10 mM GSH and 1 mM H_2_O_2_. The quantitative analysis of the fluorescence intensity after the intravesical instillation of the Cy7.5, Cy7.5 labelled ^SS^CB_GB_ and ^CC^CB_GB_ convincingly confirmed the excellent mucoadhesive property of the nanocarrier (Figure 9d). According to the results of the in vivo antitumor efficacy experiments, both ^SS^CB_GB_ and ^CC^CB_GB_ could significantly inhibit tumor growth compared with free GB or GA, which is due to the excellent mucoadhesiveness and permeability of the nanocarriers that was demonstrated. Moreover, the ^SS^CB_GB_-treated bladders had a similar appearance and average weight to the healthy bladder, which means that the intravesical instillation of ^SS^CB_GB_ not only inhibited tumor growth but caused no toxicity to the normal urothelium.

#### 3.1.2. Other Polymeric Nanoparticles for Intravesical Drug Delivery

**Figure 10:**
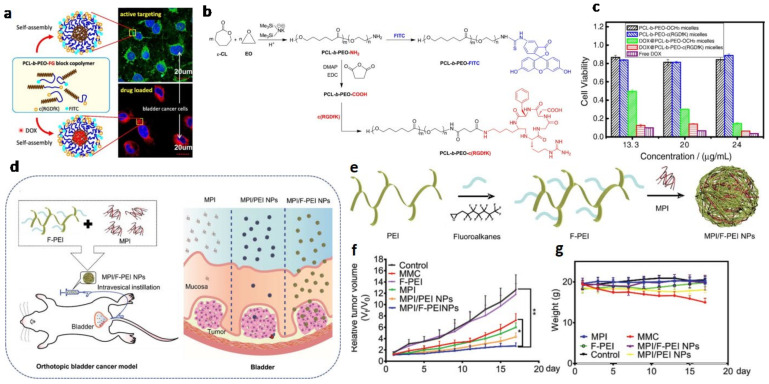
(**a**) Schematic illustration of the c(RGDfK) decorated polymeric micellar drug delivery system, which was developed for intravesical chemotherapy for superficial bladder cancer. (**b**) Synthesis of PCL-b-PEO-NH2 block copolymers and the conjugation with RGD and FITC. (**c**) Cell viability of T24 cells after incubation for 72 h with various DOX formulations in different environments. (Reprinted with permission Ref. [114]. Copyright © 2013, Elsevier.) (**d**). Schematic of (**left**) the fabrication of MPI/F-PEI NPs and their intravesical utilization in the improvement of therapeutic efficiency through (**right**) significantly ameliorating the poor bladder mucosa permeability of the MPI peptide. (**e**). The preparation of fluoroamphiphiles (F-PEI) and the fabrication of MPI/F-PEI NPs. (**f**,**g**). Tumor growth curves of mice under different treatments. (Reprinted with permission Ref. [119]. Copyright © 2019 WILEY-VCH Verlag GmbH & Co. KGaA, Weinheim.).

Due to their high drug loading capacity, controlled release profiles, and long circulation time, amphiphilic block copolymer-assembled polymeric micelles show considerable advantages as drug delivery systems for cancer therapy [110,111]. It has been demonstrated that a polymer conjugated with a cyclic (Arginine-Glycine-Aspartic acid-D-Phenylalanine-Lysine) peptide (c(RGDfK)) presented remarkable activity against prostate and endothelial cancer cell lines in vitro [112]. In addition, the conjugates efficiently enhance tumor uptake and can achieve selective therapeutic delivery to tumor sites [113]. Based on previous research, Zhou et al. reported the application of a c(RGDfK) decorated polymeric micellar drug delivery system for intravesical chemotherapy for superficial bladder cancer (Figure 10a) [114]. Poly(ethylene oxide) (PEO) and poly(ε-caprolactone) (PCL), which are FDA-approved polymers, are safe to be used in the human body, and are used for the synthesis of the PCL-b-PEO diblock copolymers that are self-assembled into micelles. A new synthetic route was used for the introduction of functional groups, the amino groups (–NH_2_) and the carboxylic group (–COOH), into the end of the PEO block. These functional groups enable the copolymer to be further conjugated with the cyclic c(RGDfK) peptide and the fluorescence molecule (Figure 10b). The c(RGDfK) modified micelles exhibited a high-affinity interaction with T24 cells, which is a kind of bladder cancer cell line. The in vitro cytotoxicity test not only indicated that the drug-loaded micelles with c(RGDfK) showed a strong inhibitory effect on T-cell proliferation, but the micelles themselves had no evident influence on cell viability, which pointed to the biocompatibility and safety of the carrier for clinical use (Figure 10c).

Besides the delivery of conventional small-molecule chemotherapeutic drugs such as doxorubicin (DOX), macromolecule drug delivery has also been attained. As an antibiotic peptide, polybia-mastoparan (MPI) is isolated from the social wasp Polybio Paulista’s venom [115]. Previous research had indicated that MPI exhibits potent antiproliferative activity against many bladder cancer cell lines, and also shows low toxicity to nontumorigenic cells [116,117]. MPI could be a highly effective, selective, relatively safe, and well-tolerated chemotherapeutic for bladder cancer intravesical chemotherapy compared to conventional chemotherapy agents [116,117,118]. However, due to the hydrophilicity and relatively high molecular weight, the transmucosal penetration of MPI could be difficult, which limits the therapeutic application of MPI in the intravesical chemotherapy of bladder cancer. To solve this problem, Li et al. used fluorinated polyethyleneimine (F-PEI), which can deliver proteins with significant advantages to deliver the MPI for intravesical instillation (Figure 10d) [119]. For the fluorophilic effect, hydrogen-bonding, and electrostatic interactions between the MPI peptide and F-PEI, they formed MPI/F-PEI nanoparticles (MPI/F-PEI NPs) by self-assembly (Figure 10e). The results indicated that the MPI/F-PEI NPs exhibited the capacity for cross-membrane, transmucosal and intratumoral penetration, due to the exclusive properties of the fluorinated chains. The therapeutic efficacy was evaluated using a suBCutaneous T24 tumor model; it is obvious that the tumor growth of MPI/F-PEI NPs treated mice was much slower than in other mice (Figure 10f) while there is no appreciable body weight loss of the MPI/F-PEI NPs treated mice (Figure 10g). This work presented a novel nanomedicine strategy, which is quite promising for the intravesical chemotherapy of bladder cancer.

### 3.2. Photothermal Therapy for Bladder Cancer

Except for the nanocarrier-based chemotherapies, there are also other nanomedicine-specific therapies (Table 4). Photothermal therapy (PTT) is a treatment method in which drugs with high photothermal conversion are exposed to near-infrared (NIR) light and release thermal energy to trigger the thermal ablation of tumors. Liu et al. constructed a Fe(III)-doped polyaminopyrrole nanoparticle (FePPy-NH_2_ NPs) to work as a photothermal converter for photothermal therapy due to its considerable ability to absorb infrared light [120]. Feppy-NH_2_ NPs are considered to have a blood circulation half-life of 7.59 h and a photothermal conversion efficiency of 44.3%, which indicates that they can accumulate around the tumor and release enough heat energy to kill tumor cells under infrared light irradiation. Gao et al. used a nanogel (NanoDOX) coated with doxorubicin (DOX) and single-walled carbon nanotubes (SWCNTs) to investigate the effect of this nanomedicine in the photothermal therapy of bladder cancer [121]. SWCNTs in this drug system were considered to have a good photothermal conversion ability and, combined with its switchable size, this nanomedicine was considered to have a great prospect for bladder cancer photothermal therapy. According to the results of the tumor-targeting experiment in mice, under near-infrared irradiation, the targeting efficiency of the nano-drug is significantly increased.

**Figure 11:**
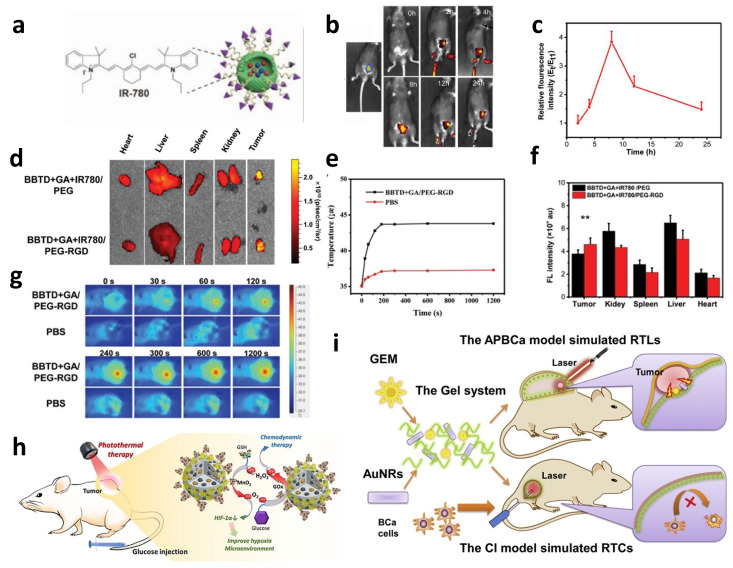
(**a**) Schematic of fluorescent BBTD+GA+IR780/PEG-RGD nanoparticles. (**b**) In vivo tumor, fluorescence imaging after BBTD+GA+IR780/PEG-RGD nanoparticles injected into the vein. (**c**) Changes in tumor fluorescence after BBTD+GA+IR780/PEG-RGD nanoparticles were injected into the vein. (**d**) Ex vivo fluorescence images of diverse tumors and organs. (**e**) Image of tumor temperature over time under 808 nm laser irradiation. (**f**) Fluorescence intensities of different organs. ** *p* < 0.01 for group of BBTD+GA+IR780/PEG compared with group of BBTD+GA+IR780/PEG-RGD in tumors. (**g**) Whole mice bodies infrared thermal images after 808 nm laser irradiation (0.8 W cm^−2^, 20 min). (Reprinted with permission Ref. [123]. Copyright © 2021 Wiley-VCH GmbH). (**h**). Schematic of chemodynamic therapy and glucose/glutathione co-triggered tumor hypoxia relief. (Reprinted with permission Ref. [124]. Copyright © 2021 American Chemical Society.) (**i**) Schematic of the single instillation of the gel system. (Reprinted with permission Ref. [125]. Copyright © 2018 Elsevier B.V.).

Due to the possible damage that high-intensity excitation light can bring to other tissues in the body, reducing the intensity of the excitation light used is one of the key points in the current research on photothermal therapy. Chen et al. developed an antibody fragment(anti-EGFR) conjugated gold nanoparticle (GNP), which was used as a probe in the process of targeting tumor cells [122]. Distinguished from other drugs used in PTT, the GNPs emit considerable heat when exposed to green light (532 nm). That means the GNPs require much less energy compared to normal drugs. Another focus of research on PTT is to find materials with higher photothermal conversion efficiency. Zeng et al. used the internal charge transfer of twisted molecules to design a molecule, 2DMTT-BBTD, which is thought to significantly improve the photothermal conversion ability [123]. The IR780 staining results of the BBTD+GA+IR780/PEG-RGD nanoparticles are shown in Figure 11a. The nanoparticles were injected intravenously into mice and accumulated to the maximum value after 8 h (Figure 11b,c). In vitro organ imaging showed that the fluorescence intensity was high within the tumor, indicating that the particles had better aggregation in the tumor cells (Figure 11d). According to the fluorescence of the BBTD+GA+IR780/PEG-RGD nanoparticles and BBTD+GA+IR780/PEG nanoparticles, it can be seen that the targeting effect of RGD contributes to the accumulation of nanoparticles (Figure 11f). Temperature monitoring under continuous laser irradiation showed that the steady-state temperature was reached after 3 min of irradiation and did not change significantly within 20 min (Figure 11e,g). The experimental results show that the photothermal conversion efficiency of this molecule to near-infrared light is up to 74.8% under 808 nm laser irradiation. This means the molecule could have the desired therapeutic effect at lower laser energies, causing less damage to the normal tissue cells surrounding bladder cancer cells.

However, the effect of single PTT in bladder cancer treatment is not always ideal, because the penetration ability of the laser is limited, and tumor cells located in deeper places cannot be destroyed. Thus, PTT is normally carried out with the assistance of other therapies. One of the adjuvant therapies is thermodynamic therapy (CDT). CDT induces apoptosis of cancer cells by making H_2_O_2_ release ·OH with its strong oxidation ability under catalytic conditions through the Fenton reaction. Chen et al. developed a therapy that enhances PTT via CDT based on glucose (Figure 11h) [124]. The nanoparticles GO_X_@MBSA-PPy-MnO^2+^ play the role of the photothermal agent in PTT by virtue of its high photothermal conversion efficiency and the release of Mn^2+^ ions under light excitation. This ion catalyzes the conversion of H_2_O_2_ to hydroxyl radical (•OH) and O_2_ through a Fenton-like reaction and kills cancer cells effectively. This combination therapy has a double-elimination effect on bladder cancer cells. Firstly, PTT was induced under the condition of near-infrared laser irradiation, and most tumor cells were destroyed. Secondly, Mn^2+^ released at the same time induces CDT destruction of residual tumor cells. In vitro and in vivo studies showed that combination therapy could inhibit the proliferation of tumor cells and completely eradicate them. PTT combined with chemotherapy can also achieve a good therapeutic effect. Guo et al. developed a smart SI gel system with PEGylated PAMAM and Dextran aldehyde to achieve a combination of photothermal therapy and chemotherapy (Figure 11i) [125]. This system induces photothermal therapy through gold nanorods (AuNRs), induces RTLs regression during photothermal ablation, and enhances the effect of chemotherapy by prolonging the drug’s action time and promoting drug accumulation. This smart gel system provides a “divide and conquer” strategy to control RTCS and RTLS, and has strong targeting and specificity for tumor cells, as well as good development prospects.

**Table 4:** Summary of the other nanomedicine-specific bladder cancer therapies.

No	Technique	Material System	Route of Administration	Advantage	Biosafety	Ref.
**1**	PTT	FePPy-NH2 NPs	intravenous injection	(a) considerable ability to absorb infrared light (b) accumulates around the tumor	good biosafety	Liu et al. (2022) [121]
**2**	PTT	NanoDOX and SWCNTs	intravenous injection	(a) high photothermal conversion efficiency (b) increased targeting efficiency	low adverse effect	Gao et al. (2017) [122]
**3**	PTT	anti-EGFR conjugated GNPs	intravesical instillation	(a) less excitation energy required	reduced damage to surrounding cells	Chen et al. (2016) [123]
**4**	PTT	2MDTT-BBTD	intravesical instillation	(a) high photothermal conversion efficiency (b) better aggregation in the tumor cells	reduced damage to surrounding cells	Zeng et al. (2021) [124]
**5**	PTT	GOX@MBSA-PPy-MnO2+	intravenous injection	(a) CDT enhances PTT and has a double-elimination effect	not clear	Chen et al. (2021) [125]
**6**	PTT	smart SI gel system	intravenous injection	(a) PTT combined with chemotherapy has a double-elimination effect (b) strong targeting and specificity for tumor cells	good biosafety	Guo et al. (2018) [126]
**7**	PDT	PLZ4-nanoporphyrin	intravesical instillation	(a) smaller diameter, highly water soluble (b) preferential tumor accumulation	fewer toxicity issues	Lin et al. (2016) [78]
**8**	PDT	HAS-MnO2-Ce6 NPs	intravenous injection	(a) improves tumor hypoxic conditions (b) good bladder tumor targeting	not clear	Lin et al. (2018) [127]
**9**	SDT	CAT-TCPP/FCS NPs	intravesical instillation	(a) realization of highly efficient cross-membrane, transmucosal, and intratumoral delivery of proteins. (b) effective SDT tumor suppression (c) relieve of tumor hypoxia	no obvious side effect	Li et al. (2020) [128]
**10**	SDT	MVs/AIEgen hybrid system	intravenous injection	(a) good homologous tumor-targeting ability (b) have the ability of effectively killing cancer cells and significantly inhibiting tumor growth	tiny damage to internal organs	Duo et al. (2021) [129]
**11**	CDT+PDT	Au@Chl/Fe-CPBA	intravesical instillation	(a) inhibition of the growth of bladder cancer cells (b) consumption of glutathione (GSH) antioxidant molecules (c) trigger of membrane lipid peroxidation	reduced systemic toxicity	Liao et al. (2022) [130]
**12**	CDT+PTT	GOx@MBSA-PPy-MnO_2_ NPs	tumor injection	(a) converts H_2_O_2_ overexpressing in tumor cells in situ generating O_2_ and preventing tumor hypoxia (b) reduces the proliferation of bladder cancer cells and completely eradicates tumor cells both in vivo and in vitro	not clear or little	Chen et al. (2021) [125]

Note: (Photothermal therapy, PTT; Photodynamic Therapy, PDT; Sonodynamic Therapy, SDT; Chemodynamic Therapy, CDT; Fe(III)-doped polyaminopyrrole nanoparticle, FePPy-NH2 NPs; Single-walled carbon nanotubes, SWCNTs; Antibody fragment, anti-EGFR; Gold nanoparticle, GNP; low bandgap benzo [1,2-c:4,5-c′]bis([1,2,5]thiadiazole), BBTD; glucose oxidase, Dox; Fe_3_O_4_, M; Bovine serum albumin, BSA; polypyrrole, PPy; Bladder cancer-specific ligand, PLZ4; Human serum albumin, HAS; Ce6 (chlorin e6); fluorinated chitosan assembled with meso-tetra (4-carboxyphenyl) porphine-conjugated catalase, CAT-TCPP/FCS; MVs/AIEgen hybrid system, AMVs; Chl/Fe for iron chlorophyll and carboxyphenylboronic for CPBA, Au@Chl/Fe-CPBA; GOx for glucose oxidase, M for Fe_3_O_4_, BSA for bovine serum albumin, and PPy for polypyrrole, GOx@MBSA-PPy-MnO_2_).

### 3.3. Photodynamic Therapy of Bladder Cancer

**Figure 12:**
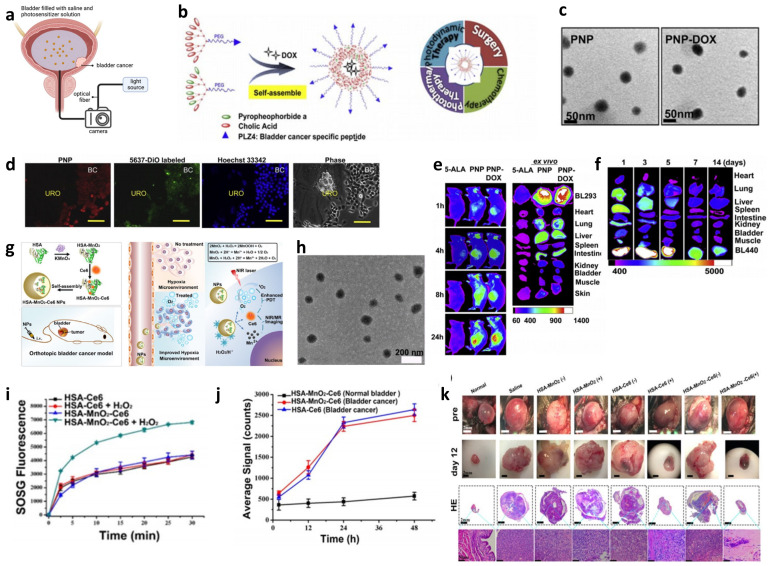
(**a**) Diagram of PDT following intravenously administered photosensitizers (Created with biorender.com). (**b**) Diagram of PNPs spontaneously assembled by the PEG^5k^-Por_4_-CA_4_ and PLZ4-PEG^5k^-CA_8_. PLZ4-micelle (PM) was a mixture of PEG^5k^-CA_8_ and PLZ4-PEG^5k^-CA_8_, while nanoporphyrin (NP) was a mixture of PEG^5k^-CA_8_ and PEG^5k^-Por_4_-CA_4_. (**c**) PNPs and PNP-DOX transmission electron microscope images. (**d**) Fluorescence microscopic examination for the selective uptake of PNPs in the typically occurring canine bladder urothelial cells (URO: no fluorescence, substantial polygonal cells with lots of cytoplasm) co-cultured with 5637 bladder cancer cells (BC: DiO pre-labelled, green). (**e**) In vivo NIFR imaging of NSG mice bearing suBCutaneous PDX BL293 up to 24 h after injection of medication: 5-ALA (100 mg/kg), PNPs (5 mg/kg), and PNP-DOX (5 mg/kg and 2.5 mg/kg). (**f**) PNP-DOX biodistribution and tumor retention at different time points after injection. (Reprinted with permission Ref. [77]. Copyright © 2016 Elsevier Ltd.) (**g**) Schematic illustration of the synthesis of HSA-MnO2-Ce6 NPs and their utilization in enhanced PDT therapy for orthotopic bladder cancer by alleviating hypoxia. (**h**) TEM image of HSA-MnO2-Ce6 NPs. (**i**) O_2_ production with HSA-Ce6 and HSA-MnO_2_-Ce6 NPs under NIR laser irradiation (660 nm laser, 5 mW/cm^2^) under an atmosphere of N_2_ in the presence or absence of H_2_O_2_. (**j**) NIR fluorescence intensity levels of bladder areas at different time intervals relying on in vivo fluorescence pictures. (**k**) HE-stained bladder sections and representative pictures of orthotopic bladder cancers before and after treatments. (Reprinted with permission Ref. [126]. Copyright © 2018 Lin, Zhao, Zhao, Yu, Cao, Chen, Wei, and Guo.).

The photodynamic phenomenon was first discovered in 1900 [130]. In 1903, this phenomenon was described as ‘photodynamic action’ by Herman Von Tappeiner and A. Jesionek when they treated skin tumors with topical eosin and white light. Photosensitizers, usually in the visible range of light, and molecular oxygen, are the three critical elements of PDT. Under the irradiation of light with a wavelength of 600–900 nm, photosensitizers will generate reactive oxygen species (ROS), which induce the cytotoxicity of cancer cells through various photochemical reactions [130]. At the same time, 100–200 mL of normal saline is often used to expand the bladder cavity to smooth the mucosal folds, and the resulting spherical light diffuser is placed in the “optical centre” of the bladder cavity (Figure 12a) [131,132].

The efficacy of PDT is closely related to the composition of the photosensitizers. Biostable photochemical properties that allow selective accumulation or retention in target tissues and minimal systemic toxicity are critical for an ideal photosensitizer [133]. So far, photosensitizer development for PDT of BCa can be roughly divided into three generations. The first generation is hematoporphyrin and its derivatives. In 1976, Kelly and Snell used a hematoporphyrin derivative to treat superficial BCa. They found fluorescence of hematoporphyrin derivatives may be useful as a diagnostic aid in some patients, and it can eliminate bladder tumors, albeit in a limited area [134]. Intravenous hematoporphyrin derivatives received the first regulatory approval for recurrent papillary bladder tumors in 1993. However, because the hematoporphyrin derivative is made up of more than 60 compounds, it is difficult to synthesize it repeatedly. Furthermore, it has certain side effects during use, such as bladder muscle damage, and the recurrence rate within one year is high, with a low treatment efficiency [134]. These shortcomings have prompted the research and development of second-generation photosensitizers. 5-Aminolevulinic acid(5-ALA) is a typical example of a second-generation photosensitizer, which is an endogenous component of heme biosynthesis and is converted into protoporphyrin IX in its active form (PPIX) [135]. When compared to first-generation photosensitizers, second-generation photosensitizers not only improve treatment efficiency, but can also be delivered orally, intravenously, or intravesically [135]. Furthermore, they do not present side effects such as decreased bladder capacity, incontinence, urinary incontinence, or worsening of the upper respiratory tract. In addition, the interval between recurrences of bladder cancer was slightly longer. However, second-generation photosensitizers still have some side effects when used, and the main disadvantage of this method is transient irritability. Furthermore, they cause obvious acute inflammation, as well as the release of various inflammatory mediators from leukocytes, and the treatment efficiency still needs to be improved [135,136].

With the advancement of nanotechnology in recent years, third-generation photosensitizers based on nanoparticles have gradually become an important factor in the photodynamic therapy of bladder cancer. To address the previous photosensitizers’ poor selectivity, low absorption band, poor bioavailability, low efficiency, and lack of photothermal effect or ability to co-deliver chemotherapeutic drugs [137], Lin et al. synthesized a multifunctional nanoporphyrin platform coated with PLZ4, a bladder cancer-specific ligand [77]. PLZ4-nanoporphyrin (PNP) combines targeted chemotherapy, photothermal therapy, image-guided photodynamic therapy, and photodynamic diagnostics into one therapy. The PNPs they designed were spherical, small (about 23 nanometers), and capable of emitting fluorescence, thermal, and active oxygen preferentially when exposed to near-infrared light, and the PNPs imaged before and after doxorubicin, (DOX) loading (PNP-DOX), were spherical (Figure 12b,c). Lin et al. conducted preclinical studies and a proof-of-principle for these novel bladder cancer-specific targeting multifunctional PNPs. According to their study, after intravesical instillation, the PNPs did not adhere to neighboring normal urothelial cells or to normal urothelial cells that were mixed with cancer cells (Figure 12d). The PNP’s activity is due to its smaller diameter (approximately 25 nm) and high water soluble, with preferential tumor accumulation, characterized by prolonged retention (up to 14 days) at the tumor site, compared to conventional photosensitizers, which have the disadvantage of poor selectivity between cancerous and non-cancerous tissues, unwanted skin accumulation leading to phototoxicity, and limited clinical applicability (Figure 12e,f). In general, the superior antitumor efficacy of PNPs can be attributed to the incorporation of three therapeutic modalities (PDT/PTT/Chemo) in one nanoformulation, as well as the unique properties of nanoplatforms. This PNP platform can be easily translated into clinical applications and may significantly improve bladder cancer management due to the fewer toxicity issues [77,138].

Another type of bladder cancer photosensitizer based on nanoparticles works by improving tumor hypoxia. The hypoxic microenvironment in bladder cancer tissue restricts the required O_2_ supply during PDT, resulting in PDT’s low efficacy in the treatment of bladder cancer [139]. Lin et al. synthesized nanoparticles with HAS (human serum albumin) as a carrier protein, MnO_2_ as a catalyst and characterization agent, and Ce6 (chlorin e6) as the photosensitizer (HSA-MnO2-Ce6 NPs) (Figure 12g,h) [126]. HAS-MnO_2_-Ce6 NPs will react with endogenous H_2_O_2_ and, thus, produce ^1^O_2_ in situ. In their experiment, the addition of hydrogen peroxide increased the ^1^O_2_ production of HSA-MnO_2_-Ce6 NPs by 2-fold under 660 nm laser irradiation, significantly improving tumor hypoxic conditions in the bladder cancer tissue and enhancing the effect of the PDT on bladder cancer cells (Figure 12i,k). Moreover, in vivo NIR imaging revealed good bladder tumor targeting of HSA-MnO_2_-Ce6 NPs (Figure 12j). Similarly, by combining Ce6-conjugated catalase (CAT-Ce6) and fluorinated polyethyleneimine (F-PEI), Li et al. created H_2_O_2_-responsive PDT nanoparticles (CAT-Ce6/FPEI) for improved PDT in bladder cancer [140].

### 3.4. Sonodynamic Therapy of Bladder Cancer

Although intravesical chemotherapy and immunotherapy are generally accepted as adjuvant treatments after surgery to avoid recurrence, these treatments still have restrictions, including the ineffective permeability of chemotherapeutic agents and dilution of the agents by urine [92]. To compensate for the defects of traditional therapy, a new therapeutic method called sonodynamic therapy (SDT) was developed, which kills malignant tumor cells by using sonosensitizers and low-intensity ultrasound (US), simultaneously [141]. The advantages of this therapy are high accuracy, deep penetration and fewer side effects [142]. However, conventional organic sonosensitizing molecules show low bioavailability and are biochemically unstable [143]. Thus, it is important to choose good sonosensitizers for the most effective and safe SDT [141]. High bioavailability and good biocompatibility are required for the ideal sonosensitizer [144], thus, testing the biosafety of sonosensitizers in SDT is critical. In terms of in vitro cytotoxicity and in vivo toxicity, a cell proliferation activity test and mouse bladder section injury observation were performed. Besides, survival curves, body weight changes of mice and stained sections of major organs were observed [127,128].

**Figure 13:**
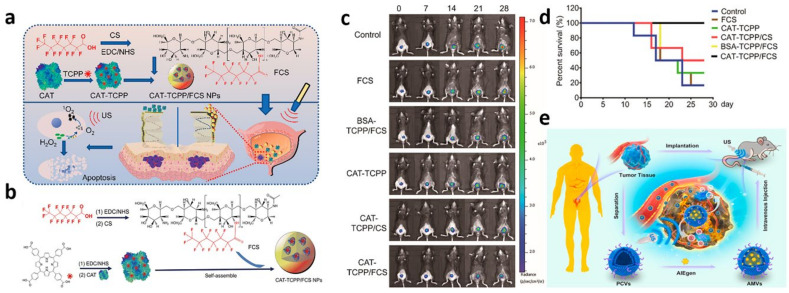
(**a**) Schematic of CAT-TCPP/FCS nanoparticles for H_2_O_2_-responsive enhanced SDT (40 kHz, 3 W/cm^2^). (**b**) Preparation of CAT-TCPP/FCS NPs. (**c**) In vivo bioluminescence images of MB49-tumor-bearing mice, which were intravesically instilled with FCS, CAT-TCPP, CAT-TCPP/CS NPs, BSA-TCPP/FCS NPs, or CAT-TCPP/FCS NPs after SDT. The bladder was exposed to ultrasonic irradiation (3.0 W/cm^2^, 15 min). (**d**) The cumulative survival rate of mice in different groups after various treatments. (Reprinted with permission Ref. [127]. Copyright © 2020 American Chemical Society.) (**e**) The schematic illustration of the patient-derived microvesicles/AIE Luminogen hybrid system for personalized sonodynamic cancer treatment in patient-derived xenograft (PDX) models. (Reprinted with permission Ref. [128]. Copyright © 2021 Elsevier Ltd.).

Li et al. have developed fluorinated chitosan (FCS) assembled with meso-tetra (4-carboxyphenyl) porphine-conjugated catalase (CAT-TCPP) to form nanoparticles for intravesical instillation SDT (Figure 13a,b) [127]. During the in vivo intravesical experiment, the fluorescence detection indicated that CAT-TCPP/FCS NPs possesses an excellent transmucosal absorption capacity and the combination with FCS significantly enhanced the penetration of CAT-TCPP into the interior of the tumor tissue. Besides, the DCFH-DA fluorescence (Figure 13c) shows that CAT-TCPP/FCS NPs could decompose intracellular H_2_O_2_ to generate ROS efficiently, which could relieve tumor hypoxia and enhance SDT efficacy. It is worth noting that CAT-TCPP/FCS shows tiny damage to the H&E-stained sections of the major organs and bladders, while CAT-TCPP/CS causes the early death of mice (Figure 13d), and the fluoroalkane modification of chitosan (CS) greatly reduces the cytotoxicity of polymers [127].

Another novel sonosensitizer used for intravenous injection SDT has also been developed. Duo et al. constructed a patient-derived MVs/AIEgen hybrid system (AMVs) and found that AMVs have an excellent tumor targeting ability and efficient personalized SDT therapy performance on a PDX model (Figure 13e) [128]. They couple microvesicles, which were from patient-derived cancer cells, and DCPy (AIEgen molecule) through electroporation. In ESR and OH detection, DCPy can produce singlet oxygen and hydroxyl radical simultaneously and exhibits superior ROS generation. In in vivo experiments, according to biodistribution evaluation detection, AMVs exhibited the most effective tumor tropism in PDX-bearing mice and possessed the best tumor targeting at different time points. Besides, the combination between AMVs and US irradiation not only exhibits great tumor growth inhibition but also causes tiny damage to the internal organs of mice [128].

### 3.5. Chemodynamic Therapy of Bladder Cancer

As an emerging and alternative cancer strategy to previous and traditional therapies, chemodynamic therapy (CDT) is based on the reactive oxygen species (ROS) transforming hydrogen peroxide (H_2_O_2_) into the hydroxyl radical (•OH) via Fenton or Fenton-like reactions [145,146]. In a specific tumor microenvironment, acidity and overproduction of hydroxyl peroxide present the essential conditions for CDT [147,148]. The Fenton reaction is defined as the generation of hydroxyl radical (•OH) from hydrogen peroxide (H_2_O_2_) catalyzed by ferrous ion (Fe^2+^), which destroys the proteins, lipids, and nucleic acids in tumor cells [124,148]. Furthermore, CDT is equipped with high logicality, high selectivity, and low toxicity compared with traditional tumor therapies. In addition, CDT can be activated by an endogenous stimulus to produce ROS, inducing cell death [148]. Therefore, CDT arouses the interest of scientists and has been widely advanced due to these merits.

Even though CDT has numerous benefits, its clinical development is hampered by its comparatively weak therapeutic effects [146]. The presence of reducing substances in the tumor microenvironment, especially glutathione (GSH) [147], reacting with the metal ion for Fenton or Fenton-like reactions may partially mitigate the effect. Therefore, exploiting novel materials for catalysts for Fenton or Fenton-like reactions, or applying CDT-based combined cancer therapy, could be solutions for those disadvantages [146,149].

Iron-based inorganic materials and other metal-based inorganic materials are two major nanomaterials for this therapy. Iron-based inorganic material is a type of nanomaterial-based on ferrous ions (Fe^2+^), generating the amount of the catalyst ion for Fenton reactions to enhance CDT efficiency in the tumor’s microenvironment. Zhang et al. synthesized and introduced a rapidly ionized and highly specific nanomaterial, which are amorphous Fe^0^ nanoparticles (AFeNPs), in an acid tumor microenvironment to release more Fe^2+^ for CDT compared with traditional Fe^0^ nanoparticles (FeNPs) [148]. Apart from ferrous ions, other transition metal ions also contribute to CDT as catalysts for Fenton-like reactions in the tumor microenvironment [145]. In the tumor microenvironment, the reduced substance glutathione (GSH) consumes the hydroxyl radical (•OH) resulting in a negative impact on CDT. Therefore, balancing the reduction of GSH and the increase of hydroxyl radicals in the tumor microenvironment is crucial. Herein, Lin et al. presented MnO_2_-based self-reinforcing CDT nano agents with both Fenton-like reaction Mn^2+^ delivery and GSH depletion capabilities [150]. The nanomaterials, MnO_2_-coated mesoporous silica nanoparticles (MS@MnO_2_ NPs), perform a redox reaction with GSH to produce glutathione disulfide and Mn^2+^. The emerging type of nanomaterial solves the challenge and provides a reference for CDT cancer therapy. A novel method to reduce toxicity was presented by utilizing CDT therapy combined with PDT therapy in this work.

**Figure 14:**
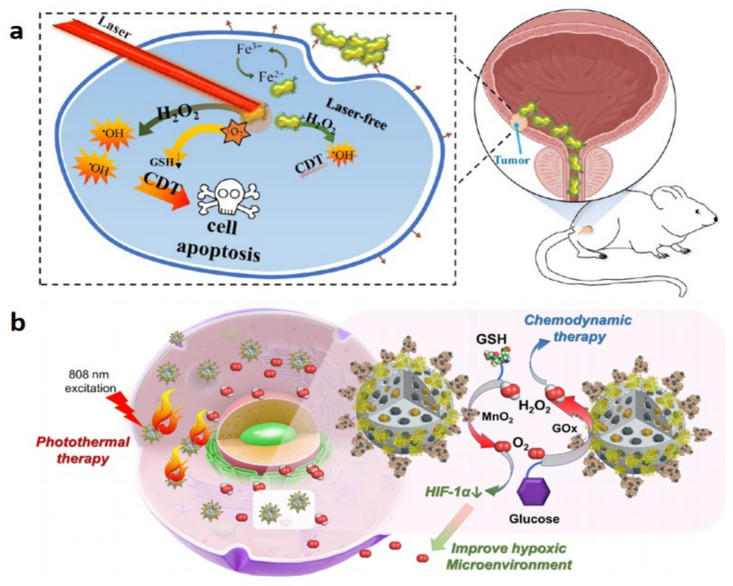
(**a**) Schematic of ROS generation enhanced by CPBA-conjugated Au@Chl/Fe nanorods by the NIR PDT process to subsequently extensively deplete GSH to produce mutual PDT and CDT in bladder cancer therapy. (Reprinted with permission Ref. [129]. Copyright © 2022 American Chemical Society.) (**b**) Schematic of the application of GOx@MBSA-PPy-MnO2 NPs in the GSH-triggered hypoxia relief of bladder cancer and efficient PTT and glucose-triggered CDT. (Reprinted with permission Ref. [124]. Copyright © 2021 American Chemical Society.).

Facing the clinical challenge of CDT, scientists prefer utilizing CDT combined with other therapies, such as PTT or PDT, to apply to bladder cancer. Liao et al. presented a CDT-based approach combined with photodynamic therapy (PDT) in which PDT excites CDT in the NIR region to treat bladder cancer (Figure 14a) [129]. The photosensitizer Au@Chl/Fe-CPBA (Chl/Fe for iron chlorophyll and carboxyphenylboronic for CPBA) reduces toxicity and avoids it from entering into systemic circulation by an intravesical instillation [129] belonging to the iron-based inorganic material. As demonstrated in Figure 14a, the experiment illustrates that the direct NIR light-induced PDT, before a lengthy CDT period, accelerates the conversion by ferrous ions in the tumor microenvironment from H_2_O_2_ to O_2_ and •OH, which exacerbates the inhibition of the growth of bladder cancer cells, resulting in the immediate consumption of the glutathione (GSH) antioxidant molecules and the triggering of membrane lipid peroxidation [129]. Localized therapy with the nanophotosensitizer provided the advantage of boosting distribution at the bladder wall and reducing systemic toxicity by preventing it from entering the systemic circulation, attributed to its delivery through intravesical instillation.

In addition, Chen et al. introduced a CDT-based combination with photothermal therapy (PTT), which is a multifunctional therapeutic platform combining PTT with glucose-triggered CDT and glutathione (GSH)-triggered hypoxia relief by tumor injection, advancing the penetrating ability of the limited laser and improving the efficiency of the H_2_O_2_ conversion (Figure 14b) [124]. The nanomaterial includes both iron-based inorganic and other metal-based material, GOx@MBSA-PPy-MnO_2_ NPs, where GOx stands for glucose oxidase; M is for Fe_3_O_4_; BSA represents bovine serum albumin, and PPy is for polypyrrole [124]. First, the thermal energy for the thermal ablation of bladder cancer is produced by the excitation of the PPy encapsulated in GOx@MBSA-PPy-MnO2 NPs. In addition, abundant H_2_O_2_ and gluconic acid are produced via the glucose catalyzed by the GOx in tumor tissues, accelerating the release of Mn^2+^. Furthermore, Mn^2+^ reacts with GSH to prevent tumor hypoxia and converts the H_2_O_2_ overexpressing in tumor cells into in situ generating O_2_. Therefore, it has outstanding photothermal efficiency for PTT as well as a Fenton or Fenton-like reaction catalyst function for CDT. In this work, PTT and glucose-triggered CDT effects and the hypoxia relief of GOx@MBSA-PPy-MnO_2_ NPs reduce the proliferation of bladder cancer cells and completely eradicate tumor cells both in vivo and in vitro. The bladder cancer therapeutic efficiency is advanced via the generation of oxygen from the materials by relieving the hypoxic tumor microenvironment [124].

As an emerging and novel cancer therapy, CDT has aroused extensive attention from scientists for its high logicality, high selectivity, and low toxicity. Although CDT therapy has the numerous merits detailed above, the clinical challenge for CDT therapy in bladder cancer is still hard to overcome. Despite the unique superiority and rapid advancement of CDT, it should be noted that CDT-based combined cancer therapy is still at the preliminary stage. There are currently just a few pieces of research and applications of CDT for bladder cancer. In other words, CDT has great possibilities and prospects in this area. The potential risks and safety issues for this type of cancer therapy when applied to bladder cancer are unknown. Furthermore, the path towards its clinical transformation is long and yet to be discovered and exploited.

Biodegradability, biosafety, biodistribution and the clearance of nanomaterials are the subject of attention and discussion. As for the nanomaterials in the treatment of bladder cancer, CDT is a novel and effective method that has already been demonstrated [146]. The majority of CDT reagents are still at the concept-proofing stage. Therefore, massive clinical translation is required to test and confirm these agents. Although the initial findings indicate that materials for CDT therapy have high biocompatibility during treatment, more work is required to increase their long-term biosafety. Furthermore, there is a risk of long-term toxicity in vivo for most CDT medicines, which still have low degradability [151]. The utilization of ultra-small or biodegradable CDT nanomaterials is a feasible way to ensure biosafety in the future [152].

### 3.6. Other Novel Achievements in Nanomedicine for Bladder Cancer Therapy

**Figure 15:**
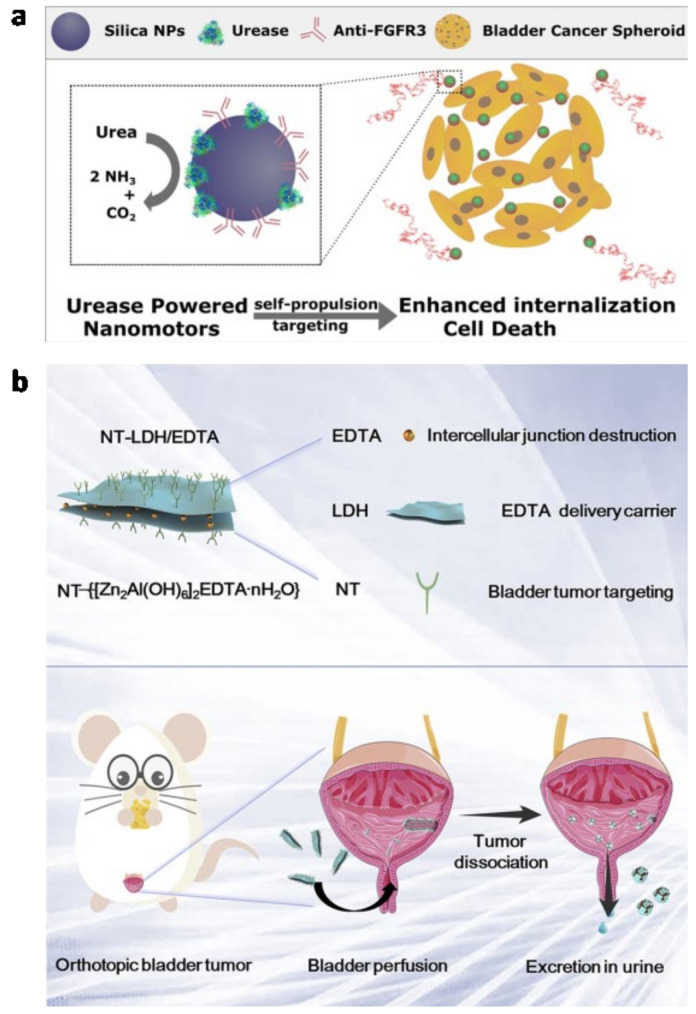
(**a**) Schematic of the urease-powered antibody-modified nanomotors for bladder cancer active targeting and therapy. (Reprinted with permission Ref. [157]. Copyright © 2018 American Chemical Society.) (**b**) Schematic illustration of ethylene diamine tetraacetic acid (EDTA) loaded neurotensin (NT)-modified Zn-Al layered double hydroxide (LDH) for bladder tumor disaggregation. (Reprinted with permission Ref. [158]. Copyright © 2020 Elsevier.).

Despite the wide range of nano-systems that have been developed for the delivery of therapeutic agents, most nanodevices do not translate into clinic because of the presence of biological barriers [153]. To solve this issue, the application of self-propelled particles in nanomedicine has been given significant attention for their capability to assist in the transport process when crossing biological barriers [154,155]. For the presence of urea in the urinary bladder, where concentrations can be 300 mM [156], urease-powered nanomotors can be designed for bladder cancer therapy. Based on this, Hortelão et al. presented the fibroblast growth factor receptor 3 (FGFR3), targeting antibody-coupled urease-powered nanomotors for active target therapy in bladder cancer spheroids (Figure 15a) [157]. The antibody not only enables the nanomotors to actively bind to the FGFR3 antigen, which is over-expressed in bladder cancer cells, but blocks bladder cancer cell proliferation and leads to cell death. The penetration was enhanced 3-fold through active motion, and the internalization efficacy of the antibody-modified active nanomotors was 4-fold higher than that of active nanomotors without the antibody.

It is known that limited intratumoral drug penetration causes resistance to several therapies used for dense solid tumors, as well as the inefficacy of drug delivery into solid tumors due to various intercellular junctions and cell-cell junctions inside integrated tumor tissues [158]. Several key connexins, which play an important role in these junctions, such as desmoglein, and desmocollin, are all calcium dependent [158]. Thus, Bao and colleagues loaded a common ion chelator, ethylene diamine tetraacetic acid (EDTA), into neurotensin (NT)-modified Zn-Al layered double hydroxide (LDH), aimed at effective tumor disaggregation (Figure 15b) [158]. Through EDTA-Ca^2+^ chelation, the EDTA molecules strip Ca^2+^ ions from the intercellular calcium-dependent connexin, which causes the disaggregation of the tumor. With the modification of neuthe rotensin (NT) peptide, which is an excellent bladder cancer cell targeting agent, the nanoplatforms can selectively accumulate at the bladder tumor sites and release the EDTA molecules. Through the intravesical administration of this novel formulation, the extraordinarily safe excretion of bladder cancer cells can be achieved [158].

Nevertheless, bladder cancer could escape from the immunosurveillance and suppress the immune response by highly expressing an immunosuppressive factor. An immunosuppressive tumor microenvironment was also created [159]. To solve this problem, Chin et al. combined photodynamic therapy (PDT) with chemodynamic therapy (CDT) [159]. PDT kills the tumor cells by heat, but the poor solubility of its photosensitizers reduces the therapeutic effect. CDT-mediated ferroptosis also has the problem of low efficiency because of the consumption of H_2_0_2_. However, after the combination, CDT-PDT not only facilitated the efficiency of treatment and reduced side effects but also decreased the expression of immunosuppressive factors by reducing the activity of PD-L1 [159]. This combined therapy could also promote the production and accumulation of immune-stimulated cells and reverse the tumor immunosuppressive microenvironment to an immunostimulatory microenvironment, which could reduce the probability of tumor recurrence [159].

### 3.7. Biosafety for Nanomaterials-Based Bladder Cancer Therapy

The biosafety concern for nanomaterials-based BCa therapy can be divided into two regimes: one is the biosafety of intravenous administrated nanomaterials and the other is the intravesical administrated therapeutic agents.

To study the biosafety of intravenous administrated NPs, Cheng’s group employed blood indexes, which included the parameters related to the blood panel counts and blood biochemistry of mice compared with the control group [152]. In Zha’s research, biosafety was evaluated by testing bladder clearance efficiency. Injected NPs with a high clearance efficiency guarantee their safety for clinical and biomedical applications [151]. In addition, liver function and renal function are some of the criteria for toxicity of intravenous nanodrugs. Liu et al. analyzed serum phosphorus (P), globulin (GLO), uric acid (UA) and other major liver and kidney function indicators to prove that FePPy-NH2 NPs have good biosafety [120]. Gao et al. focused on the serum protein after 28 days post-injection and found that NanoDOX reduced the toxicity of DOX [121].

The BCG vaccine is a commonly used drug for intravesical instillation, and has a considerable curative effect on bladder cancer. To evaluate the safety of BCG intravesical instillation, Donald cited a study of 2602 patients who underwent intravesical instillation therapy in his review, which showed that <5% of patients had adverse reactions, and these adverse reactions disappeared after stopping the use of BCG. Besides, there are appropriate drugs to eliminate these adverse reactions. All these results indicate that BCG vesical perfusion has good biosafety [160].

In conclusion, currently, the clinical biosafety assessment of nanomedicines is insufficient. Compared with biological safety, the therapeutic effect of nanomedicine is more valued. There are no standardized evaluation methods, thus, publication results related to preclinical and clinical tests from different labs or hospitals are difficult to compare [160]. Besides, more indicators of biological safety should be evaluated in clinical trials, including blood, immune, neurological, reproductive, and developmental toxicity.

## 4. Conclusions

Nanomedicine has plenty of advantages in the diagnosis and therapy of bladder cancer. In this review, the recent advances in nanomedicine in bladder cancer diagnosis and therapy were summarized and discussed.

Nanomaterials-based biosensors with electrochemical methods or optical methods can be utilized for urine cancer biomarker detection with high sensitivity and specificity. Compared with the traditional standard diagnostic methods, including cystoscope or cytology, these advanced methods are believed to be more likely to achieve a point-of-care diagnosis. The most important factor is that nanomedicine-advanced biosensors for urine cancer biomarker detection are non-invasive methods, which can avoid the risk of infection in the bladder; while the conventional cystoscope may not only cause pain to the patient but the possibility of further infection. We believe the research on urine cancer biomarker detection will be promoted into translation in the future, and finally benefit a wide range of people. Additionally, nanomaterials can be applied to fabricate imaging agents or contrast agents for the biomedical imaging of BCa and significantly improve its performance, which could be helpful in diagnosis and surgery. For imaging techniques, future research will focus on multimodal imaging, which remains a gap in BCa diagnosis, nowadays.

A high level of side effects and comparably low efficacy have been the shortcomings of chemotherapy; however, the development of drug delivery systems may be helpful to solve these problems. Concerning special anatomical structures, intravesical administration can partly avoid the systemic toxicity of chemotherapy; and nanocarriers can effectively solve the deficiencies of conventional intravesical instillation, including the weak penetration ability of the urothelium and the short residence time of the therapeutic agents. Especially for the delivery of macromolecular drugs, the development of a nanocarrier-based delivery system is essential for the drug to function. Aside from conventional chemotherapy or immunotherapy, photothermal therapy (PTT), photodynamic therapy (PDT), sonodynamic therapy (SDT), chemodynamic therapy (CDT) and combination therapy have reached many achievements in bladder cancer therapy, which have been reviewed in the above sections. Urease-powered nanomotors have also been proven to have a therapeutic effect in bladder cancer; together with a nanomaterials-based solid tumor dissociation strategy, they indicate a new direction in bladder cancer therapy. Although many achievements have been realized, there are still some hurdles to overcome during the translation of nanomedicine into the clinic, including detailed research about the interaction between bladder cancer cells and nanomaterials, and the biodistribution and clearance of nanomaterials in the bladder, which is highly correlated with the safety of nanomedicine. We also found that there is still a gap in the novel nanomaterials-assisted immunotherapy of BCa; future research may focus on the immunological effects of nanomaterials in the microenvironment of the bladder, and the delivery of nanomaterials with immunological effects to improve the outcome of BCa immunotherapy compared with conventional methods.

In general, the interesting research mentioned above has revealed the broad prospects of nanomedicine in the diagnosis and therapy of BCa. The potential for these to be translated into the clinic may come in the next few decades, and brings hope of their benefits for the health of both people and patients.

## Figures and Tables

**Table 1:** Clinical trials of drugs with nanoformulations for BCa therapy in the United States of America (https://clinicaltrials.gov, access date: 4 January 2023).

NCT Number	Nanoparticles	Drug	Condition	States	Population	Sponsor/ Collaborators	Study Start	Status
NCT05519241	PLZ4-coated paclitaxel-loaded micelles (PPM)	PTX	Non-muscle-invasive Bladder Cancer	Phase 1	18 years and older	VA Office of Research and Development University of California, Davis	October 2022	Recruiting
NCT00585689	Paclitaxel albumin-stabilized nanoparticle (Nab-paclitaxel)	PTX	Bladder Cancer	Phase 2	18 years and older	University of Michigan Rogel Cancer Center Celgene Corporation	December 2007	Completed
NCT02718742	Paclitaxel Albumin-Stabilized Nanoparticle Formulation	PTX	Recurrent Bladder Urothelial Carcinoma Stage IV Bladder Urothelial Carcinoma	Phase 2	18 years and older	Mayo Clinic National Cancer Institute (NCI)	June 2016	Withdrawn

## Data Availability

Not applicable.
